# PRKAA1/AMPKα1-driven glycolysis in endothelial cells exposed to disturbed flow protects against atherosclerosis

**DOI:** 10.1038/s41467-018-07132-x

**Published:** 2018-11-07

**Authors:** Qiuhua Yang, Jiean Xu, Qian Ma, Zhiping Liu, Varadarajan Sudhahar, Yapeng Cao, Lina Wang, Xianqiu Zeng, Yaqi Zhou, Min Zhang, Yiming Xu, Yong Wang, Neal L. Weintraub, Chunxiang Zhang, Tohru Fukai, Chaodong Wu, Lei Huang, Zhen Han, Tao Wang, David J. Fulton, Mei Hong, Yuqing Huo

**Affiliations:** 1 Drug Discovery Center, State Key Laboratory of Chemical Oncogenomics, Key Laboratory of Chemical Genomics, School of Chemical Biology and Biotechnology, Peking University Shenzhen Graduate School, 518055 Shenzhen, China; 2 Vascular Biology Center, Department of Cellular Biology and Anatomy, Medical College of Georgia, Augusta University, Augusta, GA 30912 USA; 3 School of Basic Medical Sciences, Guangzhou Medical University, 511436 Guangzhou, China; 4 College of Basic Medicine, Chengdu University of Traditional Chinese Medicine, 610075 Chengdu, China; 5 Department of Biomedical Engineering, University of Alabama at Birmingham, Birmingham, AL 35294 USA; 6 Department of Nutrition and Food Science, Texas A&M University, College Station, TX 77840 USA; 7 Department of Cardiovascular Surgery, Peking University Shenzhen Hospital, 518036 Shenzhen, China

## Abstract

Increased aerobic glycolysis in endothelial cells of atheroprone areas of blood vessels has been hypothesized to drive increased inflammation and lesion burden but direct links remain to be established. Here we show that endothelial cells exposed to disturbed flow in vivo and in vitro exhibit increased levels of protein kinase AMP-activated (PRKA)/AMP-activated protein kinases (AMPKs). Selective deletion of endothelial *Prkaa1*, coding for protein kinase AMP-activated catalytic subunit alpha1, reduces glycolysis, compromises endothelial cell proliferation, and accelerates the formation of atherosclerotic lesions in hyperlipidemic mice. Rescue of the impaired glycolysis in *Prkaa1*-deficient endothelial cells through *Slc2a1* overexpression enhances endothelial cell viability and integrity of the endothelial cell barrier, and reverses susceptibility to atherosclerosis. In human endothelial cells, PRKAA1 is upregulated by disturbed flow, and silencing *PRKAA1* reduces glycolysis and endothelial viability. Collectively, these results suggest that increased glycolysis in the endothelium of atheroprone arteries is a protective mechanism.

## Abstract (web summary)

Increased glycolysis and inflammatory responses have been observed in endothelial cells exposed to disturbed flow. However, the role of endothelial glycolysis in atherosclerosis is unclear. Here the authors unveil a protective role for glycolysis by showing that endothelial deletion of Prkaa1 accelerates atherosclerosis in hyperlipidemic mice through a reduction of glycolytic metabolism.

## Introduction

Atherosclerosis is a chronic vascular disease that involves vascular cells, leukocytes, platelets, pro-inflammatory cytokines, and growth factors^1–4^. Among these multiple factors, endothelial injury and/or denudation in atheroprone arteries has been advanced as one of the major initiating factors in the development of atherosclerosis^1–4^. Blood flow at the origins of branches and curvatures of blood vessels becomes disturbed and imparts a non-uniform and irregular distribution of low wall shear stress on the underlying endothelial cells (ECs)^5^. The combination of low wall shear stress and other atherosclerotic risk factors promotes increased EC death and compromises the integrity of the endothelial barrier^6^. This is accompanied by the infiltration of lipids and leukocytes into the arterial vessel wall and, eventually, the formation of atherosclerotic plaques. A very high turnover rate occurs on the endothelium of arteries that are prone to develop atherosclerotic lesions, suggesting that intrinsic mechanisms in ECs are necessary to provide the energy necessary to repair the compromised endothelial monolayer and protect the arterial wall from the development of atherosclerosis^7^.

Endothelial proliferation primarily relies on aerobic glycolysis to generate ATP and provide the energy resources needed for fast growth^8,9^. Glycolysis generates 75–85% of the total ATP in ECs. Of this, ECs use approximately 60% of the ATP for homeostatic maintenance and 40% for proliferation^10^. Knockdown of 6-phosphofructo-2-kinase/fructose-2,6-bisphosphatase isoform 3 (PFKFB3), a critical regulator of glycolysis, dramatically suppresses angiogenesis and demonstrates the importance of glycolysis for enabling EC proliferation^8,9^. Recent studies have found that ECs exposed to disturbed flow patterns in vitro or in vivo in areas of arterial bifurcations or curvatures exhibit increased glycolysis^11,12^. An interesting observation is that accompanying the increased glycolysis is an elevated inflammatory response in ECs in vitro and in vivo^11,12^. These data are consistent with the increased glycolysis that is seen in activated inflammatory cells^13^. Inflammation has been critically linked to the development of atherosclerosis^1–4^. Thus, a crucial question for the atherosclerosis field is whether aerobic EC glycolysis, a metabolic adaption to hemodynamic stresses that are associated with both endothelial proliferation, monolayer maintenance and inflammation, plays a harmful or beneficial role in the development of atherosclerosis.

PRKA/AMPK **(**protein kinase AMP-activated; AMP-activated protein kinases) is a major cellular energy sensor and a master regulator of metabolic homeostasis^14,15^. In mammals, PRKA/AMPK exists as a heterotrimeric complex comprised of a catalytic subunit (A1 or A2) and two regulatory subunits (B1 or B2 and G1, G2, or G3, respectively)^14,15^. PRKAA2 is the predominant catalytic form of PRKA that is found in the major metabolic organs/tissues, such as the liver, muscle, and hypothalamus, while PRKAA1 is the catalytic isoform found in vascular cells and leukocytes^15–17^. Over past decades, a large body of work has emerged to support the concept that EC AMPK is a signaling molecule that maintains endothelial homeostasis and protects cells from injury and stress^17^; however, these studies have focused primarily on acute changes in signaling, and the impact of PRKA on chronic diseases such as atherosclerosis remains poorly understood. PRKAA1 is the major catalytic form of endothelial PRKA. Its role in endothelial energy biosynthesis and endothelial proliferation, as well as endothelial injury-associated diseases such as atherosclerosis, has not been studied.

Here we find that chronic exposure to disturbed blood flow can elevate the expression and activity of PRKAA1 in ECs in vitro and in vivo. Ablation of PRKAA1 in ECs eliminates adaptive glycolysis and the increased cell proliferation in response to disturbed flow. In mice, the selective loss of endothelial *Prkaa1* decreases EC glycolysis and maintenance of the endothelial monolayer and accelerates atherogenesis. Functional deficits in *Prkaa1*-deficient ECs could be rescued by a gene therapy that increased EC glycolysis and prevented mice from accelerated atherosclerosis. Thus, this study highlights an important role of the PRKAA1–glycolysis–endothelial proliferation axis in protecting mice against atherosclerosis.

## Results

### Increased expression of Prkaa1/AMPK in atheroprone ECs

**Fig. 1:**
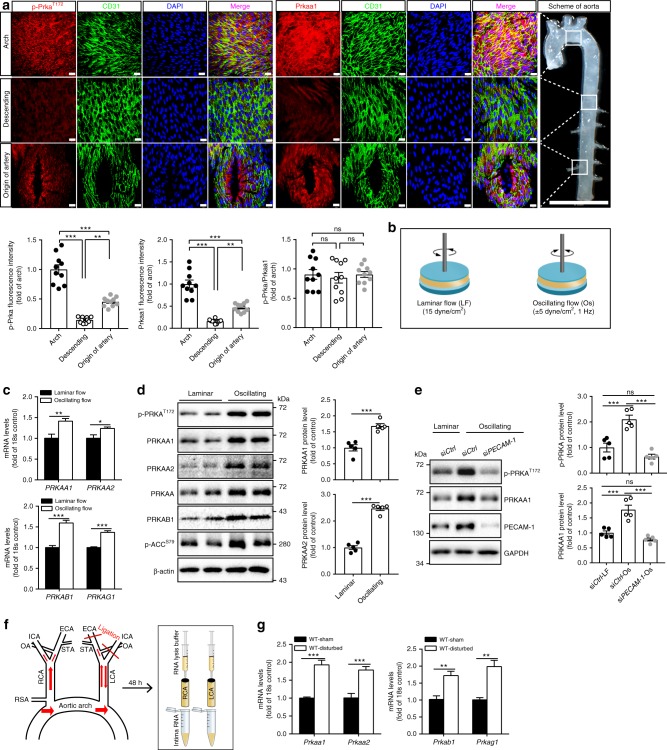
Increased expression of Prkaa1/AMPK in ECs exposed to disturbed flow. **a** Representative images of en face immunofluorescence staining and quantification data of pPrkaa1 (Thr172) and Prkaa1 (red) levels in the arterial endothelium of C57BL/6j mice. The endothelium was visualized by CD31 staining (Alexa Fluor-488, green), and nuclei were counterstained with DAPI (blue). Images were captured with confocal fluorescence microscopy. Scale bar: 20 µm; *n* = 10 mice per group. Boxes in image on the right indicate origin of regions shown in the respective rows of images (scale bar: 5 mm). **b** Schematic illustration of laminar flow (shear stress: 15 dyne/cm^2^) and oscillating flow (shear stress: ± 5 dyne/cm^2^, frequency: 1 Hz) systems in vitro. **c** Real-time PCR analysis of mRNA levels of *PRKAA1, PRKAA2, PRKAB1*, and *PRKAG1* in HUVECs under laminar flow and oscillating flow for 24 h. *n* = 4. **d** Western-blot analysis and quantification data of protein levels of pPRKA (Thr172), PRKAA1, PRKAA2, total PRKAA, PRKAB1, and pACC (Ser 79) in HUVECs under laminar flow and oscillating flow for 24 h. β-Actin was used as a loading control. *n* = 5. **e** Western-blot analysis and quantification data of the protein levels of pPRKA and PRKAA1 in HUVECs transfected with si*Ctrl* and si*PECAM-1* under laminar flow and oscillating flow for 24 h. *n* = 5. **f** Mouse partial carotid ligation model and intimal RNA extraction steps from carotid arteries following flushing arteries with QIAzol lysis reagent. **g** Real-time PCR analysis of mRNA levels of *Prkaa1, Prkaa2, Prkab1*, and *Prkag1* in ECs obtained from sham-operated right common carotid arteries and partially ligated left common carotid arteries in C57BL/6j mice. *n* = 9 mice per group. All data were expressed as mean ± SEM. Statistical significance was determined by unpaired Student’s *t*-test (for **c**, **d**, **g**) and one-way ANOVA followed by Bonferroni test (for **a**, **e**). *p < 0.05 was considered significant, **p < 0.01, ***p < 0.001

To assess the functional role of endothelial Prkaa1 in atherosclerosis, we first examined the levels of Prkaa1 and its phosphorylated form (pPrka^T172^) in the arterial endothelium of C57BL/6j mice, a strain of mice susceptible to diet-induced atherosclerosis. En face staining of the endothelium revealed increased levels of pPrka and Prkaa1 on the inner curvature of the aortic arch, an area that is prone to the development of atherosclerotic plaques, as compared to the levels observed in atheroprotective areas of the descending aorta (Supplementary Fig. 1a; Fig. 1a). ECs located at the origins of the intercostal arteries of the descending aorta, another atheroprone region, also contained much higher levels of pPrka and Prkaa1 as compared to cells more distant to these bifurcations (Fig. 1a). The ratio of pPrka/Prkaa1 in aortic arch and the origin of the intercostal arteries was similar to that in the descending aorta, indicating that increased pPrka on the endothelium of aortic arch is mainly due to the increased expression of Prkaa1 (Fig. 1a). In addition to en face staining, PRKA expression was also analyzed by western blot and reverse transcriptase (RT)-PCR assays in human umbilical vein endothelial cells (HUVECs) exposed to different flow conditions (Supplementary Fig. 1b). Oscillating flow (1 ± 5 dyne/cm^2^, 1 Hz for 24 h) markedly increased the levels of both PRKAA1 and PRKAA2 at both the protein and mRNA levels as compared to cells exposed to laminar flow (15 dyne/cm^2^ (Fig. 1b–d). Furthermore, the levels of activated forms of PRKA and scetyl-CoA carboxylase (ACC) were increased (Fig. 1d). The ratio of pPRKA/PRKAA1 in HUVECs under two flow conditions did not differ significantly (Supplementary Fig. 1c). To examine which molecule is able to transmit mechanical tension to the intracellular signaling pathways that are associated with AMPK upregulation and activation, HUVECs were treated with siRNA of *CDH5*, *PTK2*, or *PECAM-1*. Knockdown of *PECAM-1* but not *CDH5* and *PTK2* was able to significantly decrease oscillating flow-upregulated PRKAA1 expression and activation (Supplementary Fig. 1d, e; Fig. 1e).

Expression of Prka in the endothelium was also upregulated in mouse carotid arteries exposed to disturbed flow. Partial ligation of peripheral carotid artery branches generates a disturbed pattern of blood flow^18,19^ (Fig. 1f). En face staining of common carotid arteries revealed elevated protein levels of pPrka and Prkaa1 in the ECs of ligated arteries that experienced disturbed flow as compared with the levels seen in carotid arteries of sham-operated control mice (Supplementary Fig. 1f, g). The ratio of pPrka/Prkaa1 staining on both locations did not differ significantly, indicating that increased pPrka on the endothelium of ligated common carotid arteries is caused by the increased expression of Prkaa1. Assessment of changes in gene expression via real-time RT-PCR revealed that the disturbed flow pattern imposed by carotid ligation increased the mRNA expression of *Prka a1*, *a2*, *b1*, and *g1* (Fig. 1g). Collectively, these in vivo and in vitro results suggest that disturbed flow increases the expression and activation of PRKA/AMPK.

### PRKAA1 stimulates the metabolic alterations of ECs in vitro

**Fig. 2:**
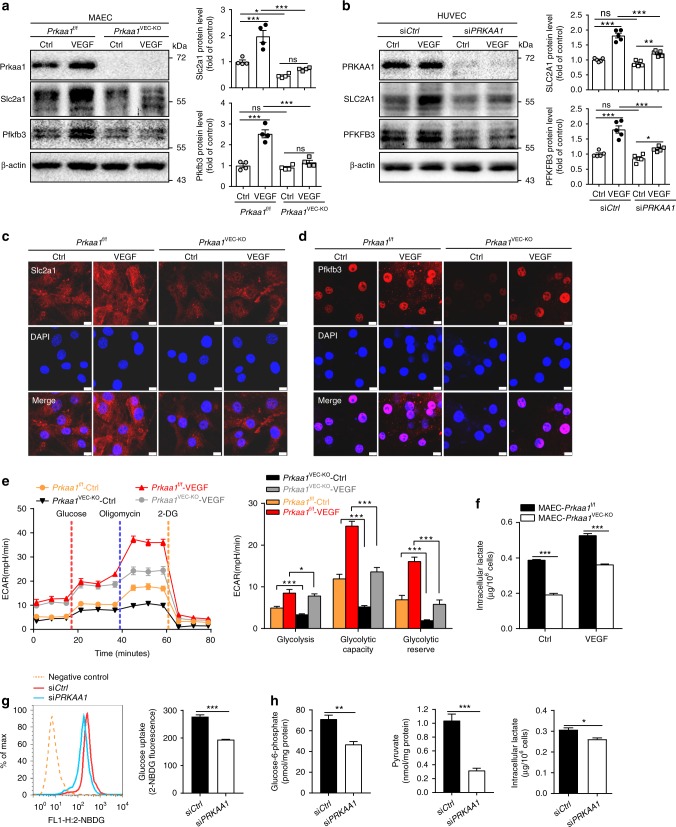
PRKAA1/AMPKα1 stimulates the metabolic alteration of ECs in vitro. **a** Western-blot analysis and quantification data of protein levels of Slc2a1 and Pfkfb3 in MAECs isolated from *Prkaa1*^f/f^ and *Prkaa1*^VEC-KO^ mice under 25% EGM-2 and VEGF 20 ng/ml 12 h treatment. *n* = 4. **b** Western-blot analysis and quantification data of protein levels of SLC2A1 and PFKFB3 in HUVECs transfected with si*Ctrl* and si*PRKAA1* under 25% EGM-2 and VEGF 20 ng/ml 12 h treatment. *n* = 5. **c**, **d** Representative images of immunofluorescence staining for cellular Slc2a1 and Pfkfb3 in MAECs isolated from *Prkaa1*^f/f^ and *Prkaa1*^VEC-KO^ mice under 25% EGM-2 and VEGF 20 ng/ml 12 h treatment. Scale bar: 10 µm; *n* = 4. **e** ECAR profile showing glycolytic function and quantification of glycolytic function parameters in MAECs isolated from *Prkaa1*^f/f^ and *Prkaa1*^VEC-KO^ mice under 25% EGM-2 and VEGF 20 ng/ml 12 h treatment. Vertical lines indicate the time of addition of glucose (10 mM), oligomycin (1 μM), and 2-DG (50 mM). *n* = 12–16 for each treatment group, replicated four times. **f** Intracellular lactate levels in MAECs isolated from *Prkaa1*^f/f^ and *Prkaa1*^VEC-KO^ mice under 25% EGM-2 and VEGF 20 ng/ml 12 h treatment. *n* = 6. **g** Representative images and quantification data of the flow cytometry analysis of 2-NBDG (100 µM, 30 min) staining in HUVECs transfected with si*Ctrl* and si*PRKAA1* for 48 h. *n* = 5. **h** Measurement of intracellular G-6-P, pyruvate and lactate levels in HUVECs transfected with si*Ctrl* and si*PRKAA1* for 48 h. *n* = 5. All data were expressed as mean ± SEM. Statistical significance was determined by unpaired Student’s *t*-test (for **f**, **g**, **h**) and one-way ANOVA followed by Bonferroni test (for **a**, **b**, **e**). *p < 0.05 was considered significant, **p < 0.01, ***p < 0.001

PRKA has been shown to regulate glycolytic metabolism in many types of cells^14,15^. To examine its role in ECs, we isolated and cultured mouse aortic ECs (MAECs) from *Prkaa1*^f/f^ or *Prkaa1*^f/f^; *Cdh5*-Cre (*Prkaa1*^VEC-KO^) mice (Supplementary Fig. 2a, b). Alternatively, HUVECs were transfected with siRNA for *PRKAA1* (si*PRKAA1*) or a control siRNA (si*Ctrl, non-targeting*). Quiescent MAECs and HUVECs were cultured in EGM2 medium supplemented with 25% of the normal growth factor levels. Exposure to VEGF-A (20 ng/ml) stimulated a significant increase in glycolysis. Expression of glycolytic or glycolysis-related genes was determined by real-time RT-PCR. Levels of hypoxia-inducible factor 1α (*HIF1A*), *SLC2A1* and *PFKFB3* mRNA were much lower in *PRKAA1* knockdown (KD) ECs than in control cells under both resting conditions and following treatment with VEGF-A (Supplementary Fig. 2c, d). Consistent with these changes, protein levels as assessed by western blot or immunostaining of cells exposed to the same conditions revealed that protein expression of SLC2A1/Slc2a1 and PFKFB3/Pfkfb3 were dramatically decreased in *PRKAA1* KD HUVECs or *Prkaa1*-deficient MAECs as compared to control cells (Fig. 2a–d). Using Seahorse Extracellular Flux analysis, we next assessed glycolytic metabolism in *Prkaa1*-deficient MAECs via measurement of the extracellular acidification rate (ECAR). As shown in Fig. 2e, *Prkaa1*-deficient MAECs exhibited significantly reduced glycolysis, glycolytic capacity, and glycolytic reserve as compared with control cells under resting conditions and increased glycolytic metabolism when stimulated with VEGF-A. Consistent with these changes, the levels of both intracellular and extracellular lactate in *Prkaa1-*deficient MAECs were much lower than those in control cells (Fig. 2f; Supplementary Fig. 2e). Levels of fructose-2,6-bisphosphate, the product of PFKFB3 and the most potent allosteric activator of 6-phosphofructo-1-kinase (PFK1), were also reduced in *Prkaa1*-deficient MAECs (Supplementary Fig. 2f). In *PRKAA1* KD HUVECs, the uptake of 2-NBDG, a fluorescently labeled glucose analog, and the level of intracellular glycolytic metabolites including glucose-6-phosphate, pyruvate, and lactate were significantly reduced compared with those in control HUVECs (Fig. 2g, h; Supplementary Fig. 2g). Altogether, these results demonstrate that PRKAA1 is an endogenous regulator of glycolysis in ECs.

### PRKAA1 is required for metabolic alterations of ECs

**Fig. 3:**
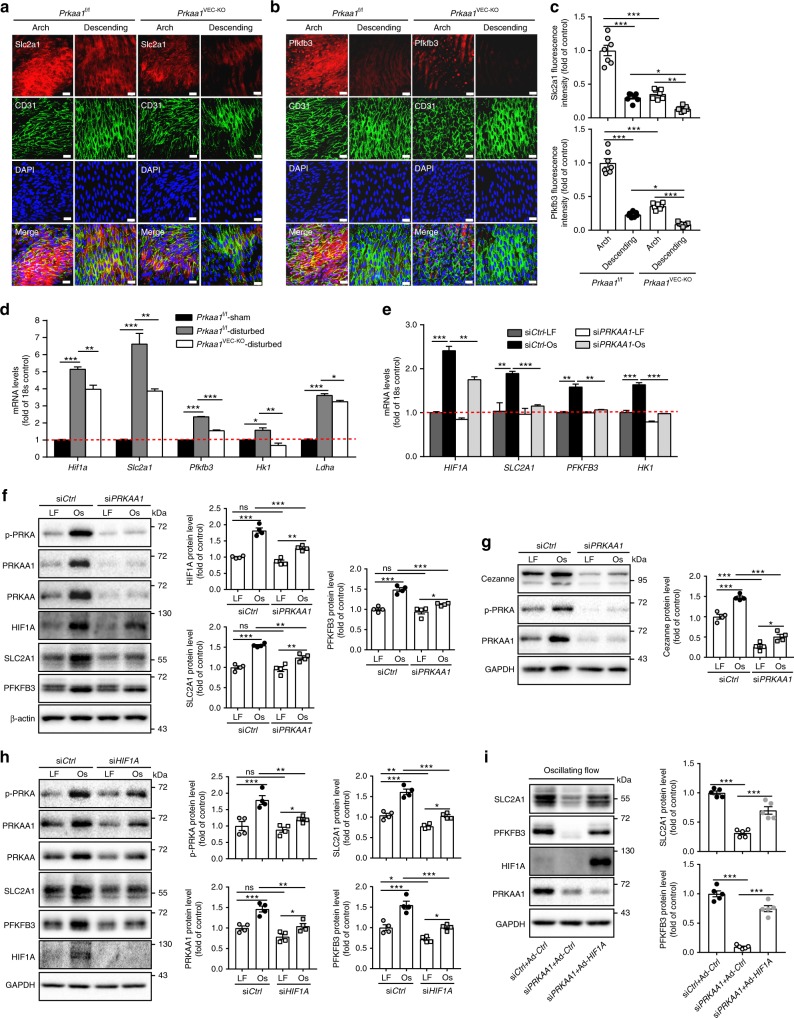
PRKAA1 is required for disturbed flow-induced metabolic alteration of ECs. **a**–**c** Representative images and quantification data of en face immunofluorescence staining for Slc2a1 and Pfkfb3 (red) on arterial endothelium of *Prkaa1*^f/f^ and *Prkaa1*^VEC-KO^ mice. The endothelium was visualized by CD31 staining (Alexa Fluor-488, green). Nuclei were counterstained with DAPI (blue). Images were captured with confocal fluorescent microscopy. Scale bar: 20 µm; *n* = 7. **d** Real-time PCR analysis of mRNA levels of glycolytic genes (*Hif1a*, *Slc2a1*, *Pfkfb3*, *Hk1*, and *Ldha*) of ECs from sham-operated right carotid arteries in *Prkaa1*^f/f^ mice and partially ligated left carotid arteries in *Prkaa1*^f/f^ and *Prkaa1*^VEC-KO^ mice. *n* = 9 mice per group. **e** Real-time PCR analysis of mRNA levels of *HIF1A*, *SLC2A1*, *PFKFB3*, and *HK1* in HUVECs transfected with si*Ctrl* and si*PRKAA1* under laminar flow and oscillating flow for 24 h. *n* = 4. **f** Western-blot analysis and quantification data of protein levels of HIF1A, SLC2A1, and PFKFB3 in HUVECs transfected with si*Ctrl* and si*PRKAA1* under laminar flow and oscillating flow for 24 h. *n* = 4. **g** Western-blot analysis and quantification data of protein level of Cezanne in HUVECs transfected with si*Ctrl* and si*PRKAA1* under laminar flow and oscillating flow for 24 h. *n* = 4. **h** Western-blot analysis and quantification data of protein levels of p-PRKA, PRKAA1, PRKAA, SLC2A1, and PFKFB3 in HUVECs transfected with si*Ctrl* and si*HIF1A* under laminar flow and oscillating flow for 24 h. *n* = 4. **i** Western-blot analysis and quantification data of protein levels of SLC2A1 and PFKFB3 in HUVECs transfected with si*Ctrl*-Ad-*Ctrl*, si*PRKAA1*-Ad-*Ctrl*, and si*PRKAA1*-Ad-*HIF1A* under oscillating flow for 24 h. *n* = 5. All data were expressed as mean ± SEM. Statistical significance was determined by one-way ANOVA followed by Bonferroni test. *p < 0.05 was considered significant, **p < 0.01, ***p < 0.001

To determine the role of Prkaa1 in regulating endothelial glycolysis in vivo, we next assessed the relative expression of glycolytic and glycolysis-related genes in the endothelium of both atheroprone and atheroprotective regions of the vasculature in mice. In line with recent publications showing that disturbed flow elevates endothelial glycolysis^11,12^, en face staining of the endothelium of the inner curvature of the aortic arch showed increased levels of Slc2a1, a glucose transporter, and Pfkfb3, whose product is an allosteric activator of 6-phosphofructo-1-kinase (PFK-1) compared with the levels seen in areas of the descending aorta that are exposed to laminar flow (Fig. 3a–c). Notably, the expression levels of Slc2a1 and Pfkfb3 in the endothelium were much lower in *Prkaa1*^VEC-KO^ mice as compared to the levels seen in the corresponding areas of control *Prkaa1*^f/f^ mice (Fig. 3a–c). In carotid arteries, endothelial expression of Slc2a1 and Pfkfb3 was upregulated by partial ligation-induced disturbed flow in control mice and was substantially reduced in vessels from *Prkaa1*^VEC-KO^ mice (Supplementary Fig. 3a, b). To more quantitatively assess these changes, the luminal region of mouse carotid arteries was liberated with QIAzol lysis reagent for EC RNA extraction at 48 h following partial ligation or sham surgery, and the mRNA expression of glycolytic and glycolysis-related genes was determined by real-time RT-PCR. The mRNA levels of *Hif1a*, *Slc2a1*, *Hk1*, *Pfkfb3*, and *Ldha* were much lower in *Prkaa1*^VEC-KO^ mice than those seen in control mice (Fig. 3d). The ability of PRKAA1 to regulate glycolytic metabolism was also observed in HUVECs subjected to disturbed flow. Exposure to oscillating flow for 24 h significantly increased expression levels of glycolytic and glycolysis-related genes at both the mRNA and protein levels, which resulted in increased lactate production, as compared to cells exposed to laminar flow (Fig. 3e, f; Supplementary Fig. 3c). However, in *PRKAA1* KD ECs, the ability of oscillating shear to increase the levels of these genes and lactate production was significantly blunted as compared to cells exposed to laminar flow (Fig. 3e, f; Supplementary Fig. 3c). These in vivo and in vitro data collectively suggest that increased glycolysis in ECs from atheroprone areas of blood vessels can be compromised through the targeted depletion of *PRKAA1*.

Since some of the above experiments showed that PRKAA1 regulated the expression of HIF1A, glycolytic and glycolysis-related genes, the interaction of HIF1A with the expression of PRKAA1, glycolytic and glycolysis-related genes was further explored. Previous study has shown that Cezanne is upstream of HIF1A in oscillating flow-mediated endothelial glycolysis^12^. In our system, Cezanne, while upregulated in control HUVECs under oscillating flow, was markedly downregulated in *PRKAA1* knockdown HUVECs, indicating that Cezanne could play a role in PPKAA1-regulated HIF1A expression in ECs under oscillating flow (Fig. 3g). In HIF1A-silencing HUVECs, the levels of p-PRKA, PRKAA1, SLC2A1, and PFKFB3 were much lower than in control HUVECs under oscillating flow (Fig. 3h). The low level of p-PRKA correlates with the decreased level of PRKAA1 in HIF1A-silencing HUVECs. To determine whether downregulated molecules following *PRKAA1* silencing are HIF1A-dependent, *PRKAA1* KD HUVECs under normoxia and oscillating conditions were transduced with Ad-*HIF1A* (active form). The suppressed expression of SLC2A1 and PFKFB3 in *PRKAA1* KD HUVECs was rescued by forced expression of HIF1A active form (Fig. 3i), indicating that these downregulated molecules in *PRKAA1* KD HUVECs are downstream of HIF1A.

### Endothelial turnover is compromised in *Prkaa1*^VEC-KO^ mice

**Fig. 4:**
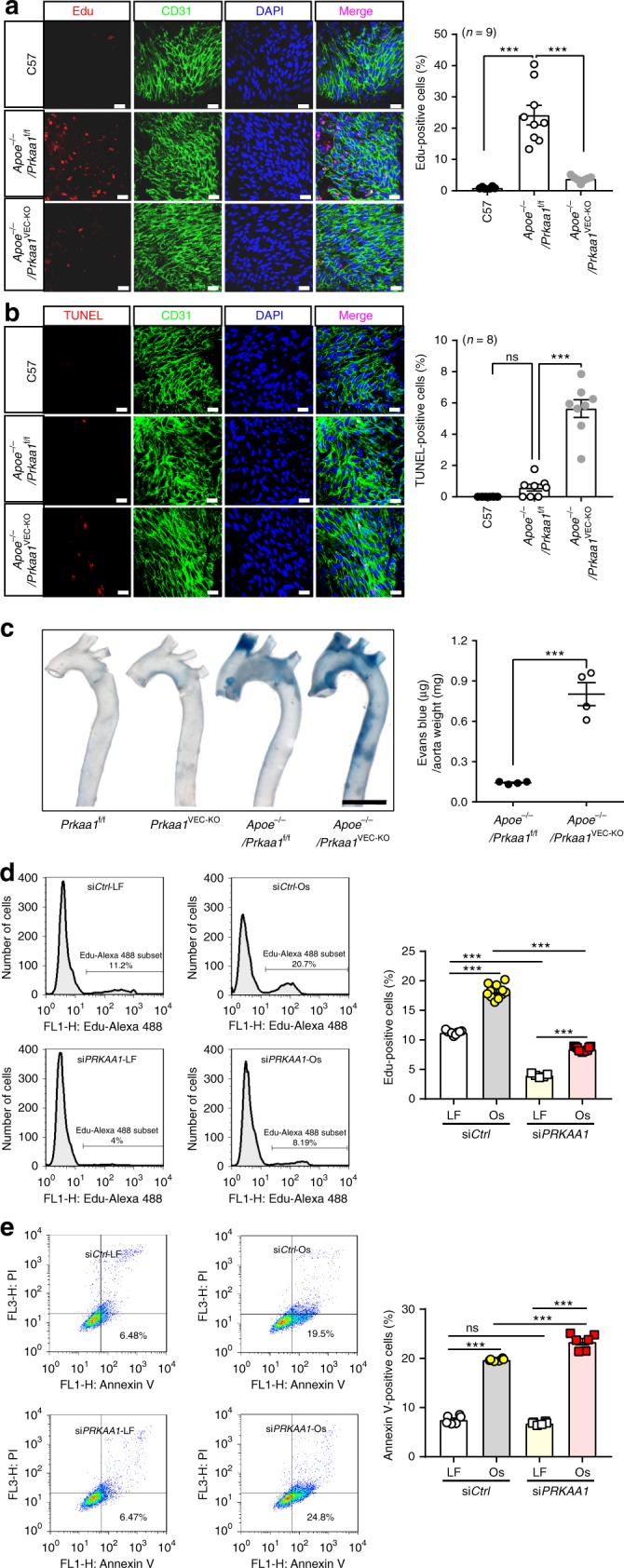
Endothelial turnover is compromised in the atheroprone areas of arteries in *Prkaa1*^VEC-KO^ mice. **a** Representative images and quantification data of en face immunofluorescence staining for Edu (red) on arch arteries in C57, *Apoe*^−/−^*/Prkaa1*^f/f^, and *Apoe*^−/−^*/Prkaa1*^VEC-KO^ mice 5 days after Edu (5 µg/g) intraperitoneal injection. The endothelium was visualized by CD31 staining (Alexa Fluor-488, green). Nuclei were counterstained with DAPI (blue). Scale bar: 20 µm; *n* = 9 mice. **b** Representative images and quantification data of en face immunofluorescence staining for TUNEL (red) on arch arteries in C57, *Apoe*^−/−^*/Prkaa1*^f/f^, and *Apoe*^−/−^*/Prkaa1*^VEC-KO^ mice. The endothelium was visualized by CD31 staining (Alexa Fluor-488, green). Nuclei were counterstained with DAPI (blue). Scale bar: 20 µm; *n* = 8. **c** (Left) Representative images of Evans blue staining of the whole aorta in *Prkaa1*^f/f^, *Prkaa1*^VEC-KO^, *Apoe*^−/−^*/Prkaa1*^f/f^, and *Apoe*^−/−^*/Prkaa1*^VEC-KO^ mice. Scale bar: 2 mm; *n* = 4. (Right) Quantification data of vascular permeability. Sample ODs of Evans blue were extrapolated to a linearized standard and normalized to weight of the aorta. *n* = 4. **d** Representative images and quantification data of flow cytometry analysis of Edu staining in HUVECs transfected with si*Ctrl* and si*PRKAA1* under laminar flow and oscillating flow for 24 h. *n* = 9. **e** Representative images and quantification data of flow cytometry analysis of Annexin V staining in HUVECs transfected with si*Ctrl* and si*PRKAA1* under laminar flow and oscillating flow for 24 h. *n* = 6. All data were expressed as mean ± SEM. Statistical significance was determined by unpaired Student’s *t*-test (for **c**) and one-way ANOVA followed by Bonferroni test (for **a**, **b**, **d**, **e**). *p < 0.05 was considered significant, **p < 0.01, ***p < 0.001

Increased endothelial proliferation occurs in atheroprone areas of blood vessels^7^. The upregulation of glycolytic programming has been well described to support the increased proliferation rates of many cell types, including ECs^8^. To investigate the role of PRKA-regulated glycolysis in endothelial turnover, we next analyzed proliferation and apoptosis of ECs from wild-type C57BL/6j, *Apoe*^−/−^; *Prkaa1*^f/f^ (*Apoe*^−/−^/*Prkaa1*^f/f^) and *Apoe*^−/−^; *Prkaa1*^f/f^; *Cdh5*-Cre (*Apoe*^−/−^/*Prkaa1*^VEC-KO^) mice. In line with observations from the groups of Tedgui and Xu^6,7^, endothelium of atheroprone areas of *Apoe*^−/−^/*Prkaa1*^f/f^ mice displayed much higher rates of Edu and TUNEL-positive staining compared to those from wild-type mice, thus revealing increased rates of proliferation and apoptosis (turnover) in the atheroprone endothelium of atherosclerotic mice (Fig. 4a, b). The apoptotic rate was further enhanced in ECs of *Apoe*^−/−^/*Prkaa1*^VEC-KO^ mice compared with those in *Apoe*^−/−^/*Prkaa1*^f/f^ mice (Fig. 4a, b). Moreover, loss of *Prkaa1* lead to dramatically decreased rates of endothelial proliferation, even in the presence of increased numbers of apoptotic cells (Fig. 4a, b). These findings indicate a mismatch in the processes required to maintain endothelial barrier integrity, which was confirmed by the enhanced permeability seen in the atheroprone vessel wall of *Apoe*^−/−^/*Prkaa1*^VEC-KO^ mice as compared to that in *Apoe*^−/−^/*Prkaa1*^f/f^ mice (Fig. 4c). Compromised proliferation in *Prkaa1*-deficient MAECs was also seen in in vitro assays in the presence and absence of VEGF-A (Supplementary Fig. 4a, b).

In cultured HUVECs, exposure to disturbed flow for 24 h increased the percentage of both Edu- and Annexin V-positive cells (Fig. 4d, e). In addition, the knockdown of *PRKAA1* further increased the number of Annexin V-positive cells but decreased the number of Edu-positive cells (Fig. 4d, e). Thus, these in vitro findings in human cells complement those found in vivo and support the overarching concept that PRKAA1 is important for endothelial turnover in atheroprone areas of the vasculature.

### Loss of endothelial *Prkaa1* increases lesion in *Apoe*^−/−^ mice

**Fig. 5:**
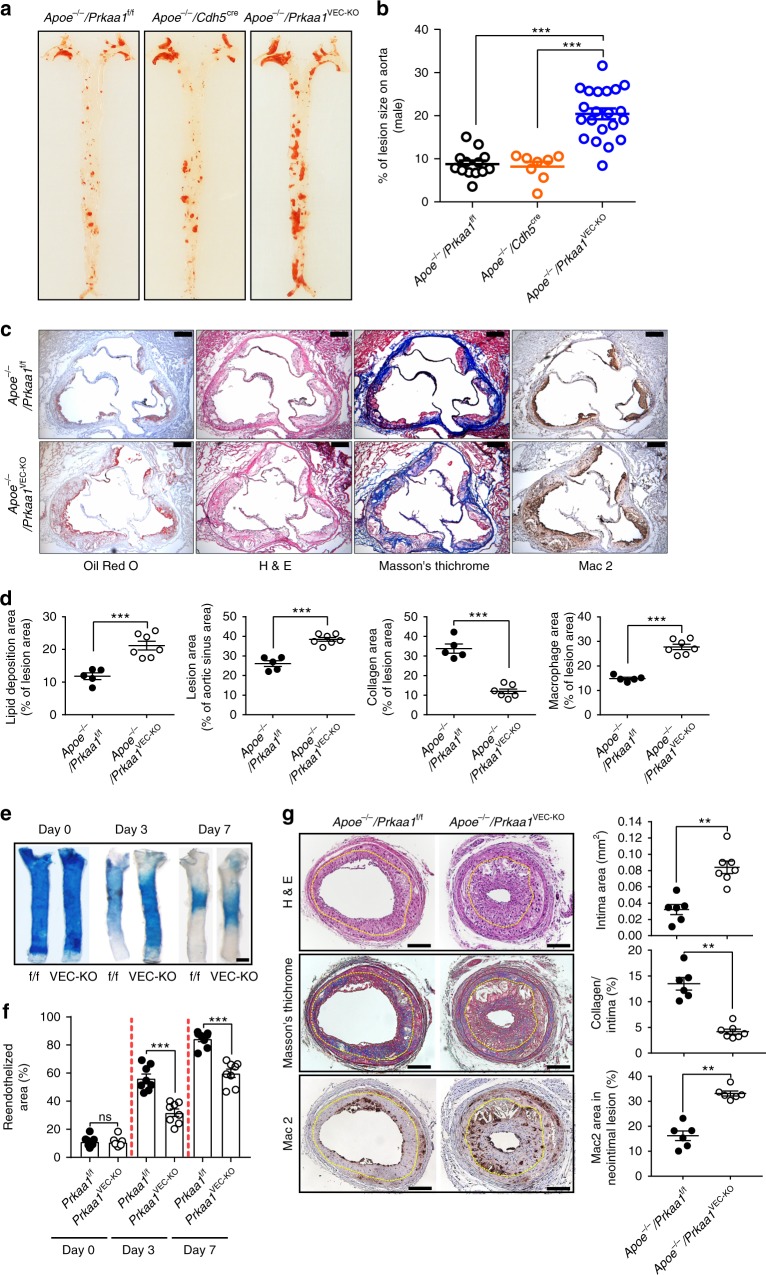
Loss of endothelial *Prkaa1* increases lesion burden in *Apoe*^−/−^ mice and accelerates neointima formation. **a**, **b** (left) Representative images of Oil Red O stained-aortas (en face) from *Apoe*^−/−^*/Prkaa1*^f/f^ (male *n* = 13), *Apoe*^−/−^ *Cdh5*^cre^ (male *n* = 8), *Apoe*^−/−^*/Prkaa1*^VEC-KO^ (male *n* = 21) mice after 16 weeks of Western diet. (Right) Lesion area quantification data. **c**, **d** Representative images and quantification data of cross-sections of aortic sinus of *Apoe*^−/−^*/Prkaa1*^f/f^ (*n* = 5), *Apoe*^−/−^*/Prkaa1*^VEC-KO^ (*n* = 7) mice with 16 weeks of Western diet. Sections were stained with Oil Red O (lesion), hematoxylin and eosin (plaque), Masson’s trichrome (collagen), and Mac-2 (a macrophage marker). Scale bar: 200 µm. **e** Representative images of Evans blue staining of injured carotid arteries harvested at the indicated time points in *Prkaa1*^f/f^ and *Prkaa1*^VEC-KO^ mice. Scale bar: 500 µm. **f** Quantification data of percentage of reendothelialization over time in the injured carotid artery from *Prkaa1*^f/f^ and *Prkaa1*^VEC-KO^ mice. *n* = 8, for each time point. **g** (Left) Representative images of paraffin cross-sections of wire-injured carotid artery from *Apoe*^−/−^*/Prkaa1*^f/f^ (*n* = 6), *Apoe*^−/−^*/Prkaa1*^VEC-KO^ (*n* = 7) mice with 4 weeks of Western diet stained with hematoxylin and eosin, Masson’s trichrome, and Mac-2. Scale bar: 100 µm. (Right) Quantification data of lesion size, collagen content, and Mac-2-positive staining. All data were expressed as mean ± SEM. Statistical significance was determined by unpaired Student’s *t-*test (for **d**, **f**, **g**) and one-way ANOVA followed by Bonferroni test (for **b**). *p < 0.05 was considered significant, **p < 0.01, ***p < 0.001

To determine whether endothelial Prkaa1 plays a role in the formation of atherosclerotic lesions, *Apoe*^−/−^/*Prkaa1*^VEC-KO^ mice, control *Apoe*^−/−^/*Prkaa1*^f/f^, and *Apoe*^−/−^/*Cdh5*-Cre mice were fed a Western diet for 4 months. There was no difference in body weight between groups (Supplementary Fig. 5a, b). Oil Red O (ORO) staining revealed a robust (2–3 times) increase in atherosclerotic surface lesion area in *Apoe*^−/−^/*Prkaa1*^VEC-KO^ mice compared to control mice (Fig. 5a, b for male mice; Supplementary Fig. 5c, d for female mice). This trend was also observed in the aortic sinus where a much larger lesion area was seen in *Apoe*^−/−^/*Prkaa1*^VEC-KO^ mice than in control mice (Fig. 5c, d). The areas of macrophage Mac-2 staining and hematoxylin and eosin (HE) staining of the necrotic core were much larger in sections of lesions from *Apoe*^−/−^/*Prkaa1*^VEC-KO^ mice than those from control mice (Fig. 5c, d). In contrast, Masson Trichrome staining of collagen in the aortic sinus was much weaker in lesions from *Apoe*^−/−^/*Prkaa1*^VEC-KO^ mice versus control mice (Fig. 5c, d). Loss of *Prkaa1* in the endothelium also increased the levels of blood glucose, triglyceride, and cholesterol as well as Ly6C^hi^ monocytes in circulating blood and spleen (Supplementary Fig. 5e–j for both male and female mice). Very likely, these factors also contribute to increased atherosclerosis in mice.

The formation of atherosclerotic lesions in wire-injured mouse carotid artery is a widely used model of accelerated atherosclerosis, and reendothelialization of the wire-injured endothelium is a key determinant of disease progression. We conducted guide-wire injury in carotid arteries and observed significant reendothelialization as determined by restoration of endothelial barrier integrity in carotid arteries. As shown in Fig. 5e, f and Supplementary Fig. 5k, the reendothelialization of carotid arteries from *Apoe*^−/−^/*Prkaa1*^VEC-KO^ mice occurred much more slowly than in control mice at various time points following injury. As a result, the size of the lesion in injured carotid arteries from *Apoe*^−/−^/*Prkaa1*^VEC-KO^ mice was significantly increased, with greater investment of macrophages and reduced collagen (Fig. 5g; Supplementary Fig. 5k, l). The above data in two models of atherosclerosis support a critical role of endothelial Prkaa1 in maintaining endothelial barrier integrity, which provides protection against the development of atherosclerosis.

### Endothelial *Slc2a1* suppresses lesions in *Prkaa1*^VEC-KO^ mice

**Fig. 6:**
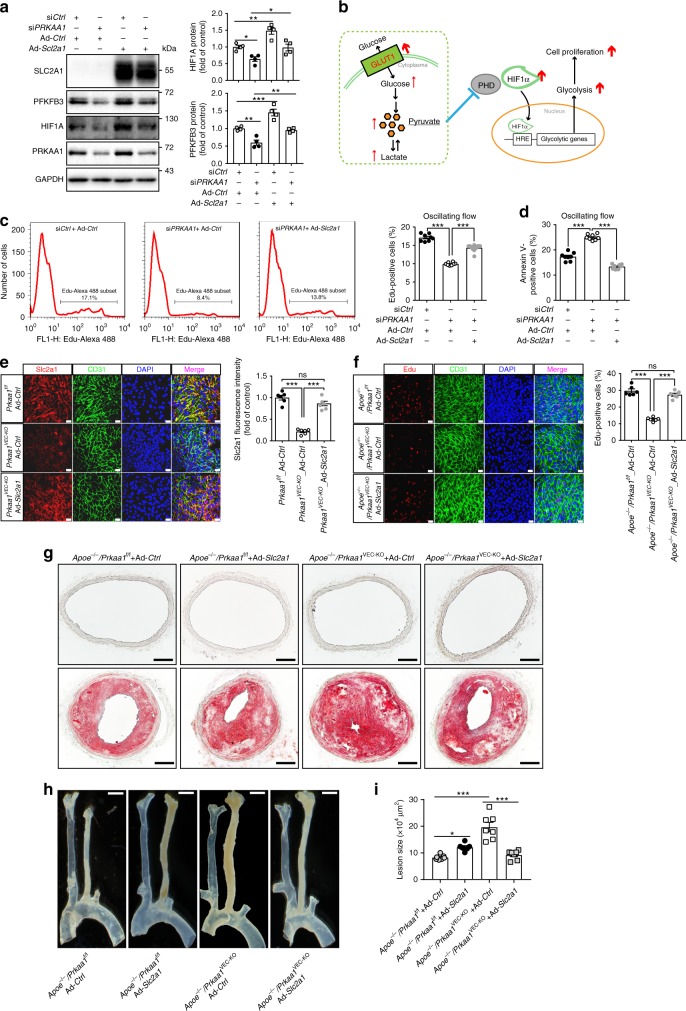
Overexpression of endothelial *Slc2a1* suppresses aggravated atherosclerosis in *Apoe*^−/−^/*Prkaa1*^VEC-KO^ mice. **a** Western-blot analysis and quantification data of protein levels of HIF1A and PFKFB3 in HUVECs transfected with si*Ctrl*-Ad-*Ctrl*, si*PRKAA1*-Ad-*Ctrl*, si*Ctrl*-Ad-*Slc2a1*, and si*PRKAA1*-Ad-*Slc2a1* for 48 h. *n* = 4. **b** Simplified schematic of rescue mechanism under overexpressing of Slc2a1. **c** Representative images and quantification data of flow cytometry analysis of Edu staining in HUVECs transfected with si*Ctrl*-Ad-*Ctrl*, si*PRKAA1*-Ad-*Ctrl*, and si*PRKAA1*-Ad-*Slc2a1* under oscillating flow for 24 h. *n* = 8. **d** Quantification data of flow cytometry analysis of Annexin V staining in HUVECs transfected with si*Ctrl*-Ad-*Ctrl*, si*PRKAA1*-Ad-*Ctrl*, and si*PRKAA1*-Ad-*Slc2a1* under oscillating flow for 24 h. *n* = 8. **e** Representative images and quantification data of immunofluorescence staining for Slsc2a1 (red) on partially ligated carotid arteries after 48 h transduction of control adenovirus (Ad-*Ctrl*) and *Slc2a1*-overexpressing adenovirus (Ad-*Slc2a1*) in *Prkaa1*^f/f^ and *Prkaa1*^VEC-KO^ mice. The endothelium was visualized by CD31 staining (Alexa Fluor-488, green). Nuclei were counterstained with DAPI (blue). Scale bar: 20 µm; *n* = 6. **f** Representative images and quantification data of Edu staining on partially ligated carotid arteries 5 days after transduction of control Ad-*Ctrl* and Ad-*Slc2a1* in *Apoe*^−/−^*/Prkaa1*^f/f^, *Apoe*^−/−^*/Prkaa1*^VEC-KO^ mice. The endothelium was visualized by CD31 staining (Alexa Fluor-488, green). Nuclei were counterstained with DAPI (blue). Scale bar: 20 µm; *n* = 6. **g**, **i** Representative cross-sections and quantification data of partially lighted carotid arteries of transduction of Ad-*Ctrl* and Ad-*Slc2a1* in *Apoe*^−/−^*/Prkaa1*^f/f^, *Apoe*^−/−^*/Prkaa1*^VEC-KO^ mice stained with Oil Red O. Scale bar: 100 µm; *n* = 7. **h** Representative photomicrographs of lesion on whole carotid arteries after transduction of Ad-*Ctrl* and Ad-*Slc2a1* in *Apoe*^−/−^*/Prkaa1*^f/f^, *Apoe*^−/−^*/Prkaa1*^VEC-KO^ mice. Scale bar: 1 mm; *n* = 7. All data were expressed as mean ± SEM. Statistical significance was determined by one-way ANOVA followed by Bonferroni test. *p < 0.05 was considered significant, **p < 0.01, ***p < 0.001

A gain-of-function approach was next used to investigate a role for “enhanced” glycolysis in the regulation of endothelial proliferation and atherosclerosis in ECs absent in *Prkaa1*. To this end, mouse *Slc2a1* adenoviral vectors were generated. Since SLC2A1 is highly conserved (>97%) between human and mouse, mouse *Slc2a1* adenoviral vectors were also used to transduce HUVECs. Optimal titers of Ad-*Slc2a1* vectors were determined from initial dose–response experiments designed to identify an appropriate viral load (5 MOI) sufficient to rescue the low level of intracellular lactate seen in *PRKAA1* KD HUVECs to a level consistent with that seen in control HUVECs (Supplementary Fig. 6a–c). Transduction of HUVECs with Ad-*Slc2a1* at 5 MOI increased glucose uptake and intracellular glycogen (Supplementary Fig. 6d–f), rescued *PRKAA1* KD-reduced expression of HIF1A and PFKFB3 (Fig. 6a, b), and collectively resulted in increased endothelial proliferation and decreased endothelial apoptosis (assayed with Edu and Annexin V staining) in *PRKAA1* KD HUVECs to the levels seen in control HUVECs (Fig. 6c, d).

To examine the role of endothelial Ad-*Slc2a1* transduction in the development of atherosclerosis, we designed a model of local viral delivery to the mouse carotid endothelium (Supplementary Fig. 7a). Using a microinjection device, 20 µl (10^8^ PFU/ml) of adenoviral vector was injected into the mouse common carotid artery where the blood flow was stopped by a gentle stretch with a suture. Blood flow was halted for 45 min to enable viral uptake. Transduction of Ad-GFP indicated viral transfection predominately occurred on the endothelium (;). A modified mouse carotid artery injury with a guide wire was first performed in a carotid artery transduced with Ad-*Slc2a1*, and an accelerated reendothelialization was observed (Supplementary Fig. 8a-c), indicating the Ad- *Slc2a1* that we used in vivo indeed increases endothelial proliferation. The Ad-*Slc2a1* was then transduced to the endothelium of mouse carotid artery, and a partial ligation was performed on the same artery to impose a disturbed flow pattern. Analysis of the endothelium or atherosclerotic lesions was conducted at 48 h or 3 weeks post procedure. Immunostaining of Slc2a1 revealed a very low level of Slc2a1 on the carotid endothelium of *Prkaa1*^VEC-KO^ mice transduced with control virus and increased expression in *Prkaa1*^VEC-KO^ mice transduced with Ad-*Slc2a1* that reached the levels seen on the carotid endothelium of *Apoe*^−/−^/*Prkaa1*^f/f^ mice transduced with Ad-*Ctrl* (Fig. 6e). Edu staining of carotid arteries showed that the rates of proliferation tracked proportionally with the level of Slc2a1 staining in the corresponding groups of mice (Fig. 6f), indicating that recovery of a low level of glycolysis in *Prkaa1*-deficient endothelium can indeed enhance cell proliferation. In the partial carotid ligation model, we found that at 3 weeks, visible atherosclerotic lesions were observed in all 4 groups of mice (Fig. 6g, h). The lesion size in *Apoe*^*−/−*^ /*Prkaa1*^VEC-KO^ mice transduced with Ad-*Slc2a1* was much smaller than lesions in the same type of mice transduced with control virus (Fig. 6g-i). In contrast, the lesion size in *Apoe*^*−/−*^*/Prkaa1*^f/f^ transduced with Ad-*Slc2a1* was larger than that in the same type of mice transduced with control virus. These results indicate that increasing glycolysis in the *Prkaa1*-deficient endothelium protects against atherosclerosis, while increasing glycolysis in *Prkaa1* intact endothelium accelerates atherosclerosis.

## Discussion

**Fig. 7:**
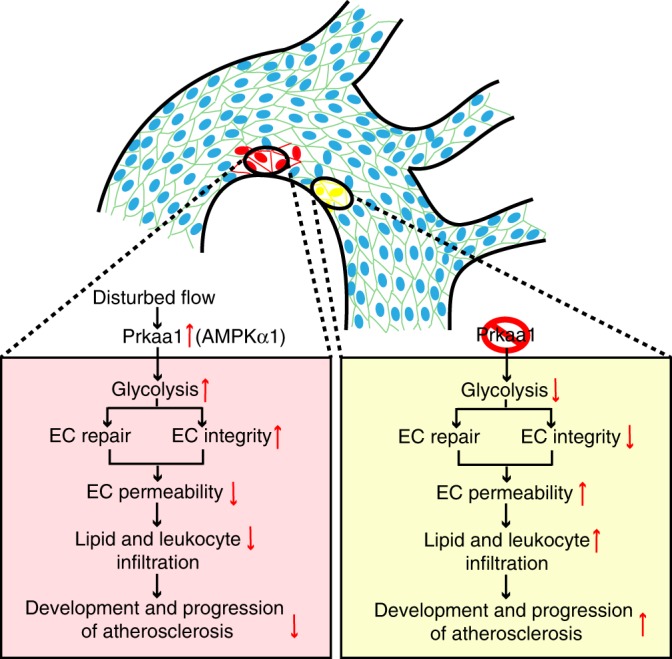
Schematic diagram illustrating the mechanisms underlying the effect of AMPKα1-mediated glycolysis on endothelial proliferation. Disturbed flow in atheroprone regions of blood vessels stimulates increased expression and activity of PRKAA1/AMPKα1 in ECs. Enhanced PRKAA1/AMPKα1 signaling promotes increased expression of HIF1A that, in turn, drives transcription of the glycolytic enzymes and consequently increased EC glycolysis. PRKAA1/AMPKα1-mediated glycolysis is vital to support increased proliferation of ECs and thus helps to preserve EC barrier integrity in vulnerable atheroprone regions and to protect from the infiltration of lipids and leukocytes into the vessel wall and the subsequent development and progression of atherosclerosis. The above protective mechanisms are compromised when the AMPKα1 pathway is blocked

Herein, we have uncovered a previously unrecognized effect of endothelial PRKAA1/AMPKα1 as a master regulator of glycolytic metabolism that is upregulated in response to pathological shear stress. Disturbed flow in atheroprone regions of blood vessels stimulates increased expression and activity of PRKAA1/AMPKα1 in ECs. Enhanced PRKAA1/AMPKα1 signaling promotes increased expression of HIF1A that, in turn, drives transcription of the glycolytic enzymes and consequently increased EC glycolysis. PRKAA1/AMPKα1-mediated glycolysis is vital to support increased proliferation of ECs and thus helps to preserve EC barrier integrity in vulnerable atheroprone regions and to protect mice from the development and progression of atherosclerosis (Fig. 7). These findings highlight the importance of endothelial PRKAA1/AMPKα1, which increases glycolysis, in the protection against atherosclerosis.

The endothelium of atheroprone regions of arteries exhibits increased expression and activity of PRKAA1. *PRKAA1* is a gene that is broadly expressed in many tissues including the kidney, liver, lung, heart, and brain^20^. It has been reported that an inverse correlation exists between the pPRKA activity level (the phosphorylated form) and aging and the severity of metabolic disorders^21,22^. The ablation of Prkaa1 in myeloid and vascular smooth muscle cells results in unfavorable pathologies in atherosclerotic arteries^23,24^. Deletion of *Prkaa2* leads to atherosclerosis^25^; however, the role of PRKAA1, the dominant PRKA catalytic subunit ECs, has not yet been investigated. Changes in the pattern of blood flow in atheroprone arteries is widely regarded as the underlying mechanism that explains the nonrandom distribution of atherosclerotic lesions in areas of turbulent flow. Laminar flow is regarded as anti-atherogenic whereas disturbed or oscillating flow is pro-atherogenic^5,26^. PRKA activation in quiescent ECs exposed to laminar flow has been observed and has been proposed as a critical mediator of the beneficial effects of laminar flow on endothelial homeostasis^27,28^. However, these studies focus on acute activation over a period of minutes. Interestingly, for the first time, our study demonstrates that the endothelium of atheroprone regions of arteries that are chronically exposed to disturbed flow have increased PRKA signaling, which is evidenced by the increased levels of pPRKA and enhanced expression levels of PRKAA1 and other PRKA family members such as PRKAA2, B1 and G1. As the catalytic subunit of PRKA, modulation of PRKAA activity (phosphorylation) has been extensively studied whereas the regulation of its expression at both the protein and mRNA levels remains underappreciated. The overall elevation of almost all PRKA family members in ECs exposed to disturbed flow may be due to either flow-mediated outside-in signaling or an intrinsic signaling reaction specific to certain flow-mediated cellular responses, for example, proliferative responses^5,26,29^. In this study, *PECAM-1* KD abrogated oscillating flow-upregulated expression of PRKA, HIF1A, SLC2A1, and PFKFB3, indicating PECAM-1, at least partially, mediates outside-in signaling in oscillating flow-upregulated endothelial glycolysis (Supplementary Fig. 9). This study adds PRKA family members to the list of protective genes in atheroprone endothelium, which has been previously revealed by a transcript study using endothelium isolated from pig aortic arch^30^.

Endothelial PRKAA1 mediates glycolysis in ECs. PRKA is a cellular energy sensor that monitors energy status and maintains energy homeostasis under metabolic stress by activating catabolic processes to increase ATP production and inhibiting anabolic processes to suppress ATP consumption^31,32^. However, the role of PRKA in metabolic change is complex, as both negative and positive regulation of aerobic glycolysis has been reported^33,34^. Metabolism in ECs is considered to be predominantly glycolytic^8,9^. Although PRKAA1 is regarded as a major energy sensor, its role in endothelial glycolysis has not yet been described. We found that stimuli promoting EC glycolysis, including disturbed flow and treatment with VEGF-A, increase the activity and expression of PRKAA1, which is followed by the upregulation of a range of glycolytic enzymes as well as increased production of lactate. These events are dependent on PRKAA1, since knockout or siRNA depletion eliminates the changes in metabolism and indicates that PRKAA1 is a critical mediator of inducible glycolytic metabolism in ECs under oscillating flow conditions.

PRKAA1-regulates endothelial glycolysis, in part via the HIF1A pathway. HIF1A is a master transcription factor that promotes tissue angiogenesis and cellular energy homeostasis under hypoxia conditions^35^. Increased HIF1A expression promotes glycolysis, resulting in a rapid supply of ATP^35^. In recent studies on endothelial glycolysis in response to disturbed flow, HIF1A has been shown to be a critical determinant^11,12^. PRKAA1 can facilitate HIF1A signaling in a multitude of ways. In DU145 human prostate cancer cells, PRKAA1 activity is required for HIF1A transcriptional activity and expression of HIF1A-targeted genes, but this does not occur through the modulation of HIF1A protein expression, stabilization, or nuclear translocation^36^. In a mouse hindlimb ischemic model, HIF1A expression in neutrophils and downstream expression of growth factors is attenuated in *PRKAA2*-deficient cells^37^. In ECs, HIF1A expression at both the mRNA and protein levels is decreased in the absence of *PRKAA1* and vice versa. A recent study revealed that Cezanne, an molecule upstream of HIF1A^12^, can also be regulated by PRKAA1. Additionally, KLF2, which is downregulated by disturbed flow and directs expression of glycolysis-related molecules, may also participate in the interactions of PRKAA1 with HIF1A^38^. Obviously, the mechanistic link and functional interaction between HIF1A and PRKAA1 appear complex and require further in-depth study. Nevertheless, there is no doubt that PRKAA1 and HIF1A are components of a concerted cellular response that regulates aerobic glycolysis in ECs.

PRKAA1-mediated endothelial metabolic homeostasis protects against the development of atherosclerosis. Increased glycolysis in atheroprone regions of arteries has been observed in multiple animal models of atherosclerosis for decades^39–41^. However, the functional role of this metabolic adaptation in atherosclerosis has remained enigmatic due to limitations in techniques and animal models. Hemodynamic stress in atheroprone areas of blood vessels is thought to compromise the EC function that triggers a cascade of events that culminates in the development of atherosclerotic lesions. An adaptive response in these regions is the fast turnover of ECs that replaces dead or dysfunctional cells. The ability to effectively maintain the EC monolayer has been regarded as a key to prevent atherosclerosis^7^. Recent studies have demonstrated a crucial role of glycolysis in the regulation of EC proliferation^8,9^. Loss or depletion of *PRKAA1*/*Prkaa1* in ECs results in a low level of glycolysis as well as accelerated atherosclerosis whereas the rescue of impaired glycolysis in *Prkaa1*-deficient ECs through the overexpression of *Slc2a1* restores endothelial proliferation and protects against accelerated atherosclerosis. These data suggest that glycolysis-supported EC proliferation and maintenance of the monolayer are critical events that prevent the development of atherosclerosis. Of note, previous studies have suggested that endothelial proliferation at disease-prone sites compromises barrier function and is an initiator of atherosclerosis^42,43^. This view is supported with studies that inhibition of angiogenesis suppresses atherosclerosis^44,45^. In our experiment on mice subjected to partial ligation of mouse carotid artery, *Slc2a1* transduction to the endothelium with intact *Prkaa1* increased the size of atherosclerotic plaques in mouse carotid arteries, indicating that hyper-glycolysis-induced excessive proliferation may trigger permeability and consequently accelerate atherosclerosis. However, endothelial proliferation mediated by PRKAA1-induced glycolysis is a cellular event in response to wounding. The extent of endothelial growth is able to maintain endothelial homeostasis but may not cause excessive proliferation, consequently decrease injury-induced endothelial permeability and protect against atherosclerosis.

PRKAA1/AMPKa1-medaited metabolic homeostasis and selective PRKA activation benefit vascular health through different mechanisms. Selective activation of PRKA with specific PRKA activators exhibits beneficial effects on cardiovascular functions^17^. Most of these effects are attribute to an increase in nitric oxide^46,47^ and may not involve glycolysis and glycolysis-associated proliferation. Indeed, in our study, we have found that both AICAR and A769662 decrease lactate production and decrease Edu-positive HUVECs, indicating that pharmacological activation of PRKA reduces endothelial glycolysis and proliferation (Supplementary Fig. 10a–c). This actually is nicely consistent with the inhibitory effect of PRKA activation on the mTOR pathway^31,48^. In contrast, *PRKAA1* KD, paralleling with its anti-proliferative effect on ECs, decreases protein synthesis of ECs (Supplementary Fig. 10d, e). The correlation of PRKAA1 protein levels with the levels of de novo protein synthesis does not indicate participation of the mTOR pathway. Thus, the current study reveals that basal PRKA and its associated glycolysis exert an anti-atherogenic effect via maintaining endothelial integrity while pharmacological PRKA activation protects the cardiovascular system via increasing nitric oxide production and/or mTOR inhibition^17,31,48^.

Endothelial HIF1A and PRKAA1 play complex roles in atherosclerosis. Previous studies have shown that disturbed flow enhances endothelial proliferation at atheroprone sites via activation of HIF1A-driven glycolysis and that deletion of endothelial *HIF1A* reduces atherosclerosis^11,12^, indicating HIF1A is a driver of atherosclerosis. In the current study, we demonstrate that the PRKAA1–HIF1A signaling axis in ECs leads to enhanced glycolysis and increased proliferation which protects mice from atherosclerosis. In this setting, the HIF1A pathway appears to have anti-atherogenic actions which reveals a greater degree of complexity than previously appreciated. In our study, the different actions of HIF1A most likely arise from the following possibilities: (i) PRKAA1 promotes homeostatic levels of endothelial proliferation that are vital in repair, whereas HIF1A may drive excessive proliferation leading to leakiness/cholesterol influx. (ii) The relationship between endothelial glycolysis and atherosclerosis may be more complex. Whether glycolysis is protective or pathogenic may be context-dependent, i.e. may vary according to age, cholesterol levels, systemic inflammation and other factors. (iii) PRKAA1 and HIF1A have pleiotropic effects unrelated to glycolysis that also influence atherosclerosis. The effect of Prkaa1 deletion on plasma cholesterol levels, circulating monocytes and others may be important in atherosclerosis, although the Slc2a1 rescue experiment has assessed the effects of PRKAA1-driven glycolysis.

The atheroprotective effect of increased endothelial glycolysis does not supersede the beneficial effect of anti-inflammatory approaches in atherosclerosis. The inflammation hypothesis for atherosclerosis has been well accepted for decades in animal models and has been recently validated in humans with the effectiveness of an IL-1β antibody in preventing cardiovascular disease^49,50^. Recent studies from Mutlu and Evans have shown that increased EC glycolysis also elevates the level of endothelial inflammation^11,12^. This is further supported by a large body of data showing that ECs in atheroprone areas of blood vessels have a heightened level of inflammation and more recent data showing increased glycolysis in the same region. A surprising observation of our study was that mice with decreased EC glycolysis developed aggravated atherosclerosis. These data suggest that the benefits of increased glycolysis and endothelial proliferation, at least in atheroprone areas, is paramount for providing protection against lesion development. The potential negative consequences of metabolic support for a chronic, low level of endothelial inflammation within this paradigm remains to be determined, and it is likely that strategies targeting the enhanced inflammation may provide even more protection against atherosclerosis. Furthermore, our data do not support recent suggestions that targeting glycolysis might be an effective strategy to prevent EC inflammation and atherosclerosis. While it may be effective against the former, it may exacerbate the latter, and therefore caution should be exercised in pursuing strategies to suppress glycolysis for cardiovascular and other proliferative diseases.

Many other mechanisms may be also involved in PRKAA1-mediated endothelial hemostasis. Reduction of fatty acid oxidation impairs de novo nucleotide synthesis for DNA replication and consequent proliferation for ECs^51^. PRKA activation increases fatty acid oxidation (FAO) in ECs^52^. In our *PRKAA1* KD ECs, FAO is decreased. Therefore, *PRKAA1 KD* ECs might have a decreased level of FAO, and decreased FAO may also be one of the factors reducing endothelial proliferation and accelerating atherosclerosis. In addition to metabolic dysfunction, compromised proliferation of *PRKAA1* KD ECs may be also due to unbalanced reactive oxygen species (ROS). *Prkka2* KD increases endoplasmic reticulum (ER) stress and atherosclerosis in vivo^25^. A trend of increase in ER stress, intracellular hydrogen peroxide, and superoxide occurs in *PRKAA1* KD ECs (Supplementary Fig. 11). This is possibly due to decreased antioxidant effects since the portion of glycolysis flux shunted to the pentose phosphate pathway may be reduced in *PRKAA1* KD ECs. Glycolysis flux diverted to the pentose phosphate pathway generates more NADPH and consumes intracellular ROS^53^.

Collectively, we have shown using *PRKAA1*-KD in human cells and *Prkaa1*-deficient mice that EC glycolysis plays a vital role in EC proliferation, maintenance of the EC monolayer, and the development of atherosclerosis. Recent studies have shown that in ECs, the level of inflammation parallels the extent of glycolysis, suggesting that glycolytic metabolism is closely associated with inflammatory signaling. While our studies add further to this paradigm, we also demonstrate that this is not a simple relationship and that EC glycolysis should not be regarded as the enemy of vascular health. Extensive studies will therefore be necessary to reveal the complexity of the intricate interplay between metabolic programming and inflammatory signaling. Successful navigation of these relationships may yield novel approaches that are capable of preserving glycolysis but decreasing inflammation in ECs, which may lead to better outcomes in the treatment of atherosclerosis.

## Methods

### Mouse generation and breeding

The use of mice was in accordance with the National Institutes of Health Guide for the Care and Use of Laboratory Animals and relevant ethical regulations. Mouse experiments were approved by the Institutional Animal Care & Use Committee of Augusta University. All mice used in this study were on a C57BL/6j background. *Prkaa1*-floxed mice were kindly provided by Dr. Benoit Viollet (Institut Cochin, Paris, France). *Apoe*^*−/−*^*/Prkaa1*^VEC-KO^ mice were generated by crossing *Prkaa1*^f/f^ with *Cdh5*^cre^ transgenic mice (Cat. No. 006137, The Jackson Laboratory, Bar Harbor, ME) and *Apoe*^*−/−*^ mice (Cat. No. 002052, The Jackson Laboratory). All mice were genotyped by PCR amplification of tail-clip samples. Experiments were performed with both male and female mice, and littermates were used as controls. Mice were housed in temperature-controlled cages under a 12-h light–dark cycle and given free access to water and normal chow.

### Atherosclerotic lesion analysis

*Apoe*^*−/−*^*/Prkaa1*^f/f^*, Apoe*^*−/−*^*/Cdh5*^cre^, and *Apoe*^*−/−*^*/Prkaa1*^VEC-KO^ male and female mice were fed a Western diet (Cat. No. TD88137; ENVIGO, Indianapolis, IN, USA) for 16 weeks starting at 7 weeks. Mice were then anesthetized with isoflurane, and blood was drawn from a heart puncture. Following euthanasia, mouse hearts were perfused with 10 ml of phosphate-buffered saline (PBS) (Cat. No. BP665-1; Fisher Scientific, Pittsburgh, PA, USA) and then 10 ml of 4% paraformaldehyde (PFA)/PBS (Cat. No. sc281692; Santa Cruz Biotechnology, Dallas, TX, USA) via the left ventricle. After incubation of isolated hearts and aortas in 4% PFA/PBS overnight, periadventitial fat and connective tissue were removed from aortas with fine forceps and scissors. Whole aortas were stained with 2% Oil Red O, and then opened longitudinally and placed on a plate for photography. The atherosclerotic lesions on aortas were quantified with Image-Pro Plus software (Media Cybemetics, Bethesda, MD) by an investigator who was blinded to the specific group assignment.

Additionally, the upper one-third of the heart was dissected and embedded in optimum cutting temperature compound (OCT; BDH Laboratory Supplier) and further cryosectioned into 5-μm-thick sections. Frozen sections were mounted onto Superfrost Plus Slides (Fisher Scientific, Pittsburgh, PA) and stained with hematoxylin or 2% Oil Red O (Cat. No. O0625; Sigma-Aldrich, Louis, MO, USA) to evaluate the size of necrotic cores or atherosclerotic lesions in the aortic sinus. Quantification was completed with Image-Pro Plus software (Media Cybemetics, Bethesda, MD, USA) by an investigator who was blinded to the specific group assignment. Five sections were quantified for each mouse in each cohort studied.

### Transluminal injury of mouse carotid artery

Arterial wire injury was performed as described previously^54,55^. Mice were anesthetized using an intraperitoneal injection of ketamine (80 mg/kg body weight) and xylazine (5 mg/kg) (Phoenix Scientific, Inc., St. Joseph, MO). After midline neck incision, the left external carotid artery was tied off distally, and a 0.014-inch flexible angioplasty guide wire was advanced by 1 cm along the common carotid artery via transverse arteriotomy. Complete and uniform endothelial denudation was achieved by three passes with a rotating motion. At different time points after injury, mice were further processed for different research objectives.

### Mouse carotid artery endothelial denudation and reendothelialization

As described in a previous publication^56^, to measure the reendothelialized area, anesthetized mice were injected through the inferior vena cava with 200 µl 2% Evans blue dye (Cat. No. E2129, Sigma) at 0, 3, and 7 days after transluminal injury of carotid artery injury. Five minutes following Evans blue injection, mice were perfused with PBS and 4% PFA/PBS. The injured artery was opened longitudinally, placed en face on parafilm, photographed with an Olympus SZX16 Stereo Microscope, and analyzed with Image-Pro Plus software. The Evans blue-stained luminal area indicated the area not covered with ECs. The endothelial regenerated area was calculated as the percentage of the non-blue area over the total injured luminal surface of the artery.

### Mouse model of injury-induced accelerated atherosclerosis

For this study, 7-week-old mice were fed the Western diet for ten days prior to transluminal guide wire injury and then for 3 weeks after injury. The carotid arteries were harvested from euthanized mice after perfusion and were fixed with 4% PFA/PBS. The arteries were embedded in paraffin and processed for serial cross-sections (5 μm) in a region of 900 μm from bifurcation to the common carotid artery. The sections were stained with hematoxylin and eosin. Image-Pro Plus software (version 6.0; Media Cybernetics) was used to measure the lumen area (LA), the internal elastic lamina (IEL) area, and the external elastic lamina (EEL) area on sections. The intima area was calculated by subtracting the LA from the IEL area, and the media area by subtracting the IEL area from the EEL area. Intima area/media area ratio is used to compare the neointima formations between different experimental groups.

### Mouse model of partial ligation of carotid artery

Partial ligation of the left carotid (LCA) was performed as previously described^18,19^. Briefly, mice were first anesthetized, the LCA was bluntly dissected, and three of four branches of the LCA, including the left external carotid, internal carotid, and occipital artery, were ligated with 7-0 silk (Cat. No. sut-s 103; Braintree Scientific, Inc., Braintree, MA, USA), and the superior thyroid artery was left intact. The incision was then closed with 3-0 silk (Cat. No. K832H; ETHICON, Inc., Cornelia, GA, USA). Postsurgical mice were recovered and fed the appropriate diet for different time periods as indicated in the different study protocols. The success of this model was confirmed by ultrasound observation with reversal flow in the LCA during diastole.

### Ex vivo measurement for vascular permeability

Mice were first prepared as indicated in the different study protocols. Mice were then injected with 2% Evans blue dye in saline (4 µl/g) via tail vein. Thrity minutes later, mice were euthanized and perfused with PBS. Aortas were immediately harvested, photographed, and weighed. Evans blue dye was extracted from the aortas by ultra-sonicating the artery in 50% trichloroacetic acid (4 µl/mg). Each sample was centrifuged, and the supernatant was mixed with 100% ethanol at 1:3 ratio. ODs of samples were read with a microplate reader at 620 nm (EL340 Bio-TEK instruments, Winooski, VT, USA). The OD of each sample was normalized to its weight.

### En face analysis of aortic endothelium

After euthanasia, mice were perfused with PBS followed by 4% PFA/PBS. The harvested aortas were fixed with 4% PFA at room temperature and permeabilized with 0.5 % Triton X-100 in PBS for 30 min. The aortas were blocked with 10% normal goat serum (Cat. No. 50062Z; Thermo Fisher Scientific, Waltham, MA) at room temperature for 1 h and incubated with primary antibodies against p-Prka (Thr172) (1:100, Cat. No. GTX52341; GeneTex, Irvine, CA, USA), Prkaa1 (1:200, Cat. No. GTX112998; GeneTex), Slc2a1 (1:100, Cat. No. ab115730; Abcam, Cambridge, MA, USA,), Pfkfb3 (1:100, Cat. No. ab181681; Abcam), and CD31 (1:100, Cat. No. DIA310; Dianova, Hamburg, Germany) overnight at 4 °C. Aortas were then washed three times with 0.1% Triton X-100 in PBS; incubated with the appropriate Alexa Fluor-488- or Alexa Fluor 594-conjugated secondary antibodies diluted 1:250 in blocking solution for 1 h at room temperature; washed three times again; and stained with DAPI at 1:5000 dilution at room temperature for 5 min. Images were acquired with an upright confocal microscope (Zeiss 780; Carl Zeiss). Aortas stained with only primary or secondary antibody were used as negative controls.

For en face staining with 5-ethynyl-2′-deoxyuridine (Edu, Cat. No. A10044; Invitrogen, Waltham, MA, USA), mice were first IP injected with Edu for 5 days at a dose of 5 µg/g mouse for each day. Collected aortas were incubated with Edu reaction buffer for 30 min at room temperature.

For TUNEL staining, the collected aortas were fixed, permeabilized, and stained with the In Situ Cell Death Detection Kit, TMR Red (Cat. No. 12156792910; Roche, Indiana, IN, USA) as per the manufacturer’s instruction.

### HUVECs exposed to laminar or oscillating flow in vitro

HUVECs were seeded on a 100 mm TC-Treated Cell Culture Dish (Cat. No. 353003; Corning, NY, USA) coated with 0.1% gelatin (Cat. No. G1393; Sigma) and cultured to confluence in EGM-2 medium (Cat. No. cc3162; Lonza, Basel, Switzerland). Cell dishes were set up into a cone plate flow system that sheared at 15 dynes/cm^2^ for laminar flow or ±5 dynes/cm^2^ and 1 Hz frequency for oscillating flow for 24 h. The flow system was kept at 37 °C temperature and 95% humidified air with 5% CO_2_. Cells were then washed in PBS and further processed for Western blot, RNA extraction, or flow cytometry analysis.

### Isolation of mouse aortic ECs

As described in a previous publication^57^, the collagen surface in a 24-well plate was prepared by mixing rat tail collagen type one (Cat. No. 354236; BD Bioscience, San Jose, CA, USA), 10% FBS DMEM (Cat. No. 11965-092; Thermo Fisher Scientific) and 0.1 M NaOH, and equilibrating overnight with complete medium containing EGM-2 (Lonza), 20% FBS, 1% PSF, and cAMP before aorta segments were added into the wells.

Thoracic aortas were collected from euthanized *Prkaa1*^f/f^ and *Prkaa1*^VEC-KO^ mice, and periadventitial fat and connective tissue were removed from the vessels. The aortas were opened longitudinally and cut coronally into circular segments. The segments were washed in cell growth medium to remove blood from the intra-aortic lumen and were then placed with the lumen side onto the collagen matrix-coated 24-well plates. Three to five days later, when cellular outgrowth from the aortic segments was visible, the aortic segments were removed from the collagen matrix. The matrix was digested with 1 mg/ml collagenase D (Cat. No. 11088882001; Sigma) for 10 min at 37 °C. The subsequent cell suspensions were centrifuged, and the cells were re-suspended in complete medium, reseeded onto a T25 tissue culture flask, and then cultured in EGM-2 medium at 37 °C in a humidified incubator with 5% CO_2_.

### RNA isolation and real-time PCR

Total RNA of MAECs or HUVECs were extracted using Trizol Reagent (Cat. No. 15596018; Invitrogen, Grand Island, NY). Reverse transcription was performed with iScript cDNA synthesis kit (Cat. No. 170-8891; Bio Rad, Hercules, California, USA). Samples for real-time PCR were prepared in a StepOne Plus system (Applied Biosystems) by mixing cDNAs, Power SYBR Green PCR Master Mix (Cat. No. 4367659; Life Technologies) and gene-specific primers listed in Supplementary Table 1. All reactions were done in a 10 µl reaction volume. PCR amplification consisted of 10 min of an initial denaturation step at 95 °C, followed by 40 cycles of PCR at 95 °C for 15 s, and 60 °C for 1 min. All experiments were repeated independently at least three times. Quantification of relative gene expression was calculated with the efficiency-corrected 2^−△△CT^ method using 18S (human/mouse) RNA as the internal control, and data were presented as fold change relative to control groups.

### Intimal RNA isolation from carotid arteries

Isolation of intimal RNA from carotid arteries was performed according to a method previously described^18,19^. Briefly, the anesthetized mice were perfused with PBS via the left ventricle, and then the left and right common carotid artery (LCA and RCA) were isolated and carefully removed of periadventitial tissues surrounding the carotids. In a microfuge tube a quick flush of the carotid lumen was performed with a 31-gauge syringe filled with 150 μl of QIAzol lysis reagent (Cat. No. 5346994; Qiagen, Venlo, Netherlands). The intima eluate and the rest tissue were used for intimal or media/adventitia RNA isolation using the miRNeasy mini kit (Cat. No. 217004; Qiagen). Reverse transcription was performed with iScript cDNA synthesis kit (Cat. No. 4368814; Applied Biosystems™, Foster, CA, USA).

### Western-blot analysis

MAECs or HUVECs were lysed in RIPA buffer (Sigma) supplemented with 1% protease and 1% phosphatase inhibitors (Roche) for 10 min on ice. After centrifugation of cell lysates at 4 °C for 5 min, proteins were quantified with the BCA assay and then loaded onto the 10% SDS-PAGE gel at 10–20 μg per lane. Western-blot analysis was performed using antibodies against PRKAA1 (1:1000, Cat. No. ab110036; Abcam), P-PRKA (Thr172) (1:1000, Cat. No. 2531; Cell Signaling Technology, Danvers, MA, USA), PRKAA2 (1:1000, Cat. No. ab3760; Abcam), PRKAA (1:1000, Cat. No. ab80039; Abcam), PRKAB1 (1:1000, Cat. No. 4150T; Cell Signaling Technology), p-ACC (S79) (1:1000, Cat. No. 3661S; Cell Signaling Technology), HIF1A (1:1000, Cat. No. AF1935; R&D Systems, Minneapolis, MN, USA), SLC2A1 (1:5000, Cat. No. ab115730; Abcam), PFKFB3 (1:1000, Cat. No. ab181681; Abcam), GAPDH (1:5000, Cat. No. sc47724; Santa Cruz Biotechnology), and β-actin (1:5000, Cat. No. sc47778; Santa Cruz Biotechnology). All protein levels were normalized to β-actin or GAPDH signal. Images were taken with the CheminDoc MP System (Bio Rad) and viewed in Image J software for data analysis. Uncropped scans for western blots are provided in Supplementary Figs. 12–16.

### Flow cytometry analysis

Cell proliferation and protein synthesis of HUVEC were determined by Click-iT Edu imaging kits (Cat. No. C10337; Invitrogen) and Click-iT HPG Alexa Fluor Protein Synthesis Assay Kit (Cat. No. C10428; Invitrogen) according to the manufacturer’s recommendations. Briefly, the cells were pretreated with Edu or HPG at 10 µM for 12 h, harvested from the in vitro flow system, washed with PBS twice, fixed with 4% PFA for 15 min at room temperature, and followed by permeabilization with 0.5% Triton X-100 in PBS for 20 min at room temperature. Samples were incubated with Edu or HPG reaction buffer for 30 min at room temperature. After staining, cells were washed with 1 ml FACS buffer (0.1% BSA and 0.02% NaN_3_ in PBS) and then analyzed with a FACSCalibur flow cytometer with CellQuest software (BD Pharmingen, San Jose, CA, USA).

Apoptotic cells were analyzed by fluorescein isothiocyanate (FITC) Annexin V Apoptosis Detection Kit I (Cat. No. 556547, BD Biosciences) consistent with the manufacturer’s protocol. In brief, cells collected from the disturbed flow system were washed in PBS, centrifuged, and incubated in 100 µl binding buffer containing 5 µl propidium iodide (PI) and 5 μl FITC Annexin V for 15 min at room temperature in the dark. The samples were analyzed by FACSCalibur flow cytometer within 1 h after staining. Apoptotic cells were regarded as the cells that stained positive for Alexa FITC-conjugated Annexin V and negative for PI.

### Immunohistochemistry

Paraffin blocks or frozen blocks were sectioned at 5 µm intervals using a paraffin microtome or Microm cryostat. For frozen sections, slides were washed with PBS and permeabilized with 0.5% Triton X-100 for 20 min at room temperature before destroying the endogenous peroxidase activity and blocking with avidin/biotin solution. For paraffin sections, slides were deparaffinized and rehydrated first, followed by destroying endogenous peroxidase activity in H_2_O_2_ (3 ml 30% H_2_O_2_ in 200 ml methanol) for 30 min at room temperature. Then, sections were boiled in citrate acid buffer (10 mM, pH 6.0) by microwaving at 98 °C for 10 min for antigen retrieval. After cooling down, sections were blocked with avidin blocking solution with 10% normal serum of the secondary antibodies. The sections were incubated with primary antibodies in biotin blocking solution against Mac-2 (3 µg/ml, Cat. No. ACL8942F; Accurate Chemical & Scientific Corporation, Westbury, NY) at 4 °C overnight in a humidified chamber. After aspiration of primary antibodies and washing in FSGP/PBS for 5 min at room temperature for three times, the sections were incubated with biotinylated secondary antibodies (Cat. No. BA4001; Vector Laboratories, Burlingame, CA) for 1 h at room temperature, followed with VECTASTAIN® ABC reagents (1: 200, Cat. No. PK6100; Vector Laboratories) for 30 min at room temperature and DAB solution for 2–5 min. Sections were counterstained with hematoxylin I (Cat. No. GHS116; Sigma) for 15 s at room temperature. The sections were viewed with an Olympus BX41 microscope (OLYMPUS, Shinjuku, Tokyo, Japan) with CellSens program (Olympus, Shinjuku, Tokyo, Japan).

### WST-1 proliferation assay

HUVEC proliferation was assayed with CytoSelect™ WST-1 Cell Proliferation Assay kit (Cat. No. CBA-253; Cell Biolabs Inc., San Diego, CA). Briefly, cells in an exponential phase of growth were harvested and seeded in 96-well plates at a density of 1500 cells per well. Cells were cultured for 72 h at 37 °C and 5% CO_2_ in a humidified incubator. Following the culture or treatments as indicated, 10 µl CytoSelect™ WST-1 Cell Proliferation Assay Reagent was added into each well. Plates were then incubated for an additional 2 h at 37 °C. Subsequently, absorbance was determined in a microplate reader (EL340 Bio-TEK Instruments, Wihnooski, VT, USA) at 450 nm.

### Metabolic measurements

As described in a previous publication^58^, MAECs were seeded onto Seahorse XF96 culture cell plates at a concentration of 1.5 × 10^4^ per well, and incubated with 25% EGM-2 or VEGF 20 ng/ml at 37 °C overnight. On the second day, the treatment media were removed and cultured cells were washed once with assay medium, which is XF base medium supplemented with 2 mM glutamine, pH adjusted to 7.4 with 0.1 M NaOH. The medium was switched to assay medium with treatments, and plates were incubated for 1 h in a non-CO_2_ incubator at 37 °C. Cells were then assayed with an XFe96 extracellular flux analyzer (Seahorse Bioscience). Inhibitors and activators used in the test are glucose (10 mM), Oligomycin (1 μM), and 2-DG (50 mM).

### Measurement of intracellular glucose and glycogen

The intracellular glycogen and glucose of HUVECs were measured using glycogen assay kit II (Cat. No. ab169558; Abcam) and glucose assay kit (Cat. No. ab65333; Abcam) according to the manufacturer’s instructions. The absorbance was determined in a microplate reader (EL340 Bio-TEK Instruments, Wihnooski, VT, USA) at 450 nm for glycogen and 570 nm for glucose.

### Measurement of glucose-6-phosphate, pyruvate and lactate

The levels of glucose-6-phosphate, pyruvate, and lactate of HUVECs and MAECs were determined using High Sensitivity Glucose-6-Phosphate Assay Kit (Cat. No. MAK021; Sigma), Pyruvate Assay Kit (Cat. No. MAK071; Sigma) and the Lactate Assay Kit (Cat. No. MAK064; Sigma) according to the manufacturer’s instructions.

### Measurement of NADPH level and ROS production

The NADPH level was measured using NADPH assay kit (Cat. No. KA1663; Abnova, Taipei, Taiwan) according to the manufacturer’s recommendations. This measurement is based on a glucose dehydrogenase cycling reaction wherein the formed NADPH reduces a formazan reagent. The absorbance was read at 570 nm.

For the intracellular H_2_O_2_ level and superoxide level, HUVECs were incubated with 5 μM CM-H2DCF-DA (Cat. No. C6827; Invitrogen) or 5 μM DHE (Cat. No. 601290; Cayman Chemical, Ann Arbor, Michigan, USA) for 30 min at 37 °C protected from light. Cells were washed, and the samples were analyzed with FACSCalibur flow cytometer with CellQuest software (BD Pharmingen, San Jose, CA, USA).

### Measurement of intracellular free calcium

Intracellular calcium concentration was measured as previously described^59^. Briefly, cells were pre-transduced with adenovirus encoding cytosolic aequorin at 5 MOI for 36 h, then seeded in white 96-well plate at a density of 10,000 cells/well and incubated overnight. Cells were incubated with 100 μl of modified KRB (0.5 ml of 1 M CaCl_2_ was added to 500 ml of KRB) that contained 15 μM coelenterazine (Cat. No. 303-500; NanoLight Technologies, Pinetop, AZ, USA) for 1 h at 37 °C in a 5% CO_2_ incubator. Luminescence emission representing free intracellular calcium concentrations were obtained by a POLARstar Omega spectrophotometer (BMG Labtech, Cary, NC, USA). Relative intracellular calcium was expressed as percentage of control.

### Measurement of plasma cholesterol, triglyceride, and blood glucose levels

*Apoe*^*−/−*^*/Prkaa1*^f/f^ and *Apoe*^*−/−*^*/Prkaa1*^VEC-KO^ mice were fasted for 12–14 h before blood was collected. Plasma glucose, cholesterol, and triglyceride levels were measured enzymatically using Infinity reagents (Cat. No. TR15421, TR13421, and TR22421; Thermo Scientific). Blood glucose levels were determined by testing tail blood with an OneTouch Ultra Blood Glucose Monitoring System (LifeScan Milpitas, CA).

### siRNA transduction of HUVECs

HUVECs were transfected with siRNA targeting human *PRKAA1* (*PRKAA1* siRNA (si*PRKAA1*), Cat. No. sc-29673; Santa Cruz Biotechnology) or non-targeting negative control (Control siRNA (si*Ctrl*), Cat. No. sc-37007; Santa Cruz Biotechnology,) using siRNA transfection reagent according to the Santa Cruz protocol. Briefly, the siRNAs were prepared as 10 μM stock solutions in RNAse-free water provided. HUVECs grown at 60–70% confluence in six-well plates were incubated in 1 ml of EBM-2 medium containing RNAiMax (Cat. No. 13778-150; Invitrogen) for 30 min. A 200 µl mixture with 100 µl EBM-2 containing 5 µl siRNA and 100 µl EBM-2 containing 5 µl RNAiMax was added to each well of a six-well plate. The cells were incubated with this mixture for 4 h at 37 °C, which was then replaced with EGM-2 for a continuous culture. The cells underwent different treatments within 48 h after siRNA transduction and were then collected for different assays.

### Adenovirus transduction of ECs

Mouse *Slc2a1* adenovirus (Ad-*Slc2a1*) and negative control adenovirus (Ad-*Ctrl*) were generated by Vector Biosystems Inc. (Malvern, Pennsylvania, USA). HUVECs grown at 60–70% confluence were incubated with 500 µl basal medium containing Ad-*Ctrl* or Ad-*Slc2a1* adenovirus at 5 MOI (multiplicity of infection) for 2 h, which was then replaced with fresh complete cell medium for a continuous culture. The cells were treated as indicated within 48 h after viral transfection and then collected for Western blot, lactate measurement, and flow cytometry analysis.

For adenoviral transduction to the endothelium of mouse carotid arteries, eight-week-old *Apoe*^*−/−*^*/Prkaa1*^f/f^ or *Apoe*^*−/−*^*/Prkaa1*^VEC-KO^ mice were first anesthetized. The LCA was bluntly dissected and blood flow was blocked by a gentle stretch with 7-0 silk sutures under the internal carotid, occipital carotid, and distal external carotid artery. With a 100 µl syringe (Cat. No. NANOFIL-10; World Precision Instruments, Sarasota, FL, USA) connected to a 35G blunt needle (Cat. No. NF35B-2; World Precision Instruments), 20 µl 10^8^ PFU/ml Ad-*Ctrl* or Ad-*Slc2a1* was injected into the left common carotid arteries via transverse arteriotomy on the distal external carotid artery. The carotid artery was incubated with adenoviral buffer for 45 min. The external carotid artery was ligated on the area between the arteriotomy site and the branch to the superior thyroid artery. The partial ligation of the carotid artery model was completed on the same carotid artery with adenoviral transfection to examine the effect of gene transduction on the formation of atherosclerotic lesion caused by disturbed flow.

### Statistical analysis

The optimal animal numbers and sample sizes were determined by power analysis, prior experience, and our preliminary data to achieve statistical significance. Statistical analysis was performed using GraphPad Prism (LA Jolla, CA). The significance of the differences between two groups was determined using unpaired Student’s *t*-test. For multiple comparisons, one-way analysis of ANOVA followed by Bonferroni’s post hoc tests was used. All results are shown as mean ± SEM. *p* *<* 0.05 was considered significant (*p < 0.05, **p < 0.01, ***p < 0.001). All biological experiments were repeated at least three times using independent cell cultures or individual animals (biological replications).

## Electronic supplementary material

Supplementary Information

## Data Availability

The authors state that all data are available within this article and its Supplementary Information file are available from the corresponding author upon reasonable request.
