# Supplementary material for: PRKAA1/AMPKα1-driven glycolysis in endothelial cells exposed to disturbed flow protects against atherosclerosis
Source: Nat Commun. 2018 Nov 7;9:4667. doi: 10.1038/s41467-018-07132-x (PMC6220207; doi:10.1038/s41467-018-07132-x)
Supplement: Supplementary file 1 — Supplementary Information [file 41467_2018_7132_MOESM1_ESM.pdf]

PRKAA1/AMPKα1-driven glycolysis in endothelial cells exposed to disturbed flow protects against atherosclerosis

Yang et al.

Supplementary Figures

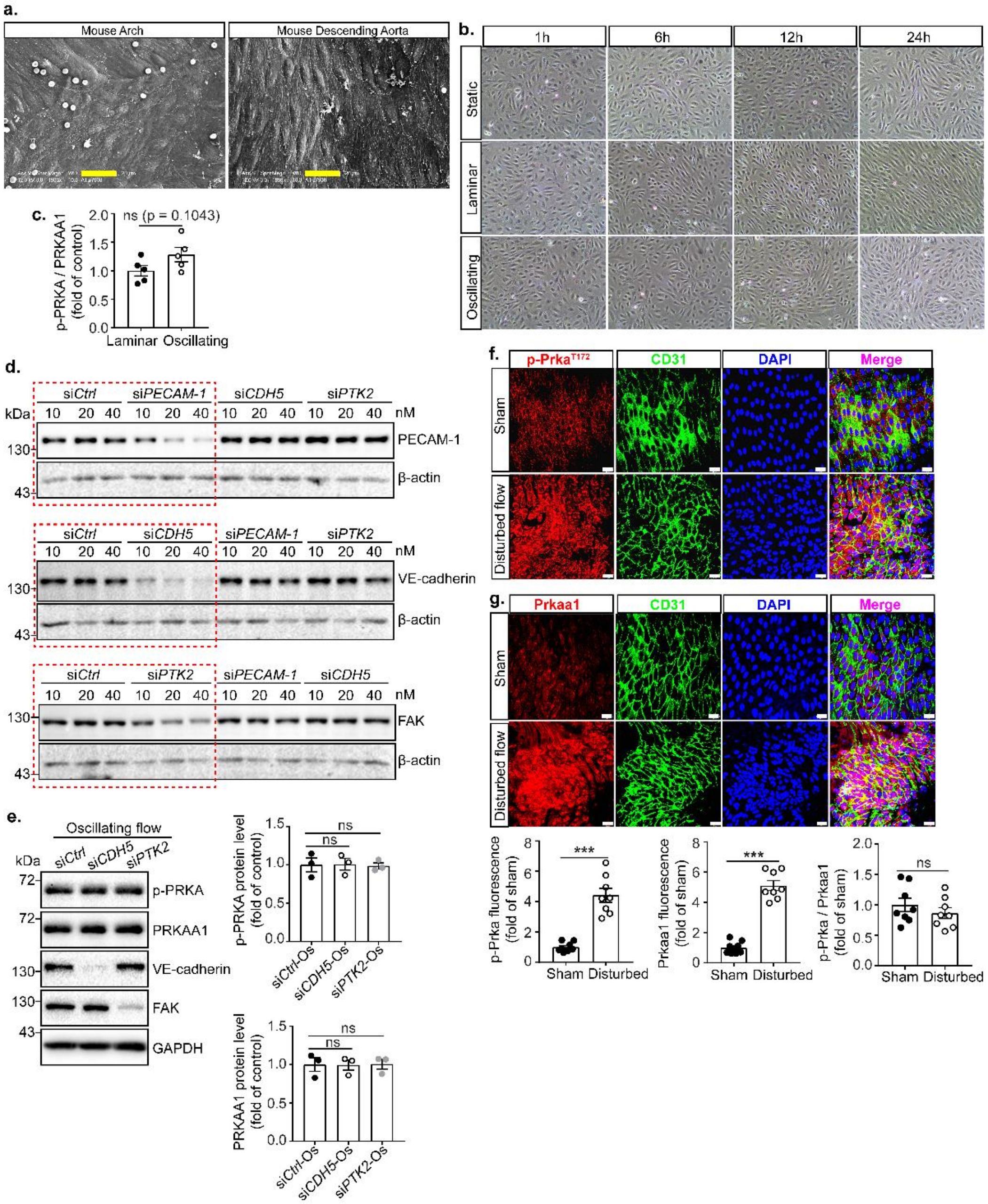

Supplementary Figure 1. Increased expression of Prkaa1/AMPK in ECs exposed to disturbed flow. **a.** The morphology of mouse arch and descending aorta endothelium under scanning electron microscopy. Scale bar: 20 μm. **b.** The morphology of HUVECs under static, laminar flow (shear stress: 15 dyne/cm^2) and oscillating flow (shear stress: ± 5 dyne/cm^2, frequency: 1Hz) systems *in vitro* at various time points. **c.** Quantification data of protein levels of p-PRKA to PRKAA1 in HUVECs under laminar flow and oscillating flow for 24h. n = 5. **d.** Western-blot analysis of protein levels of PECAM-1, VE-cadherin and FAK in HUVECs transfected with si*Ctrl*, si*PECAM-1*, si*CDH5* and si*PTK2* for 48h. **e.** Western-blot analysis and quantification data of p-PRKA and PRKAA1 in HUVECs transfected with si*Ctrl*, si*CDH5* and si*PTK2* under oscillating flow for 24h. n = 3. **f, g.** *En face* immunofluorescence staining and quantification of phosphorylated Prka (T172) and Prkaa1 in sham-operated right common carotid arteries and partially ligated left common carotid arteries in C57BL/6j mice co-stained with CD31 and DAPI. Scale bar: 20 μm; n = 8 mice per group. All data were expressed as mean ± SEM. Statistical significance was determined by unpaired Student's *t* test (for **c**, **f**, **g**) and one-way ANOVA followed by Bonferroni test (for **e**). * $p < 0.05$ was considered significant, ** $p < 0.01$, *** $p < 0.001$.

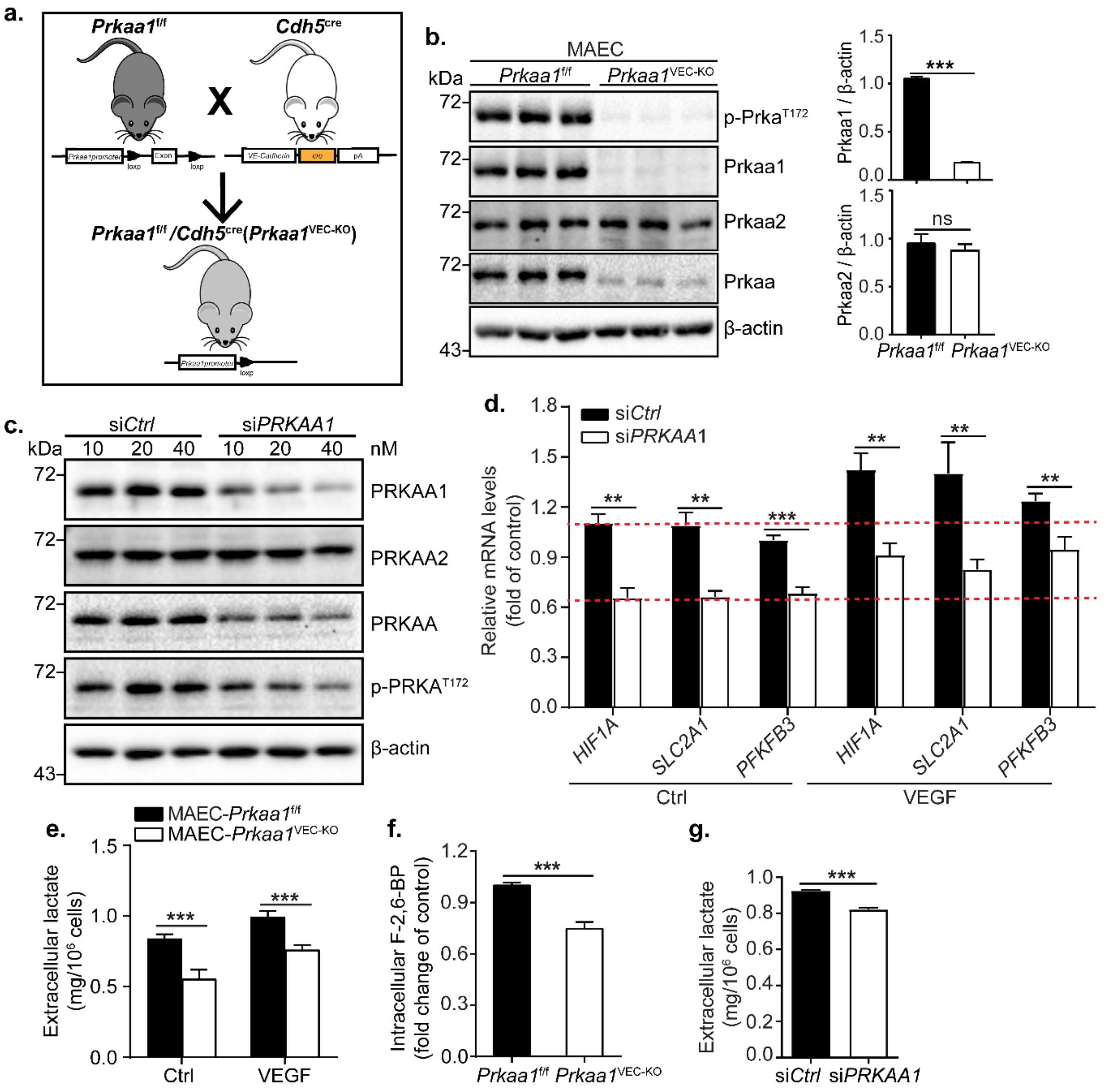

a.
Prkaa1f/f
Cdh5cre
X
Prkaa1f/f /Cdh5cre(Prkaa1VEC-KO)
b.
MAEC
kDa
Prkaa1f/f
Prkaa1VEC-KO
p-PrkaT172
Prkaa1
Prkaa2
Prkaa
β-actin
Prkaa1 / β-actin
Prkaa2 / β-actin
***
ns
c.
siCtrl
siPRKAA1
kDa 10 20 40 10 20 40 nM
PRKAA1
PRKAA2
PRKAA
p-PRKAT172
β-actin
d.
siCtrl
siPRKAA1
Relative mRNA levels (fold of control)
HIF1A
SLC2A1
PFKFB3
Ctrl
VEGF
e.
MAEC-Prkaa1f/f
MAEC-Prkaa1VEC-KO
Extracellular lactate (mg/10⁶ cells)
Ctrl
VEGF
f.
Intracellular F-2,6-BP (fold change of control)
Prkaa1f/f Prkaa1VEC-KO
g.
Extracellular lactate (mg/10⁶ cells)
siCtrl siPRKAA1

Supplementary Figure 2. PRKAA1/AMPKα1 stimulates the metabolic alterations of ECs *in vitro.* **a.** Generation of *Prkaa1*$^{VEC\text{-}KO}$ mice by crossing *Prkaa1*$^{f/f}$ mice with *Cdh5*^{cre} mice. **b.** Western-blot analysis and quantification data of protein levels of p-Prka (Thr 172), Prkaa1, Prkaa2, and Prkaa in MAECs isolated from *Prkaa1*$^{f/f}$ and *Prkaa1*$^{VEC\text{-}KO}$ mice. β-actin was used as loading control. n = 5. **c.** Western-blot analysis of protein levels of PRKAA1, PRKAA2 in HUVECs transfected with si*Ctrl* and si*PRKAA1* at different doses. **d.** Real time-PCR analysis of mRNA levels of *HIF1A*, *SLC2A1* and *PFKFB3* in HUVECs transfected with si*Ctrl* and si*PRKAA1* under 25% EGM-2 and VEGF 20ng/ml 12h treatment. n = 4. **e.** Extracellular lactate levels in MAECs isolated from *Prkaa1*$^{f/f}$ and *Prkaa1*$^{VEC\text{-}KO}$ mice under 25% EGM-2 and VEGF 20ng/ml 12h treatment. n = 6. **f**. Level of fructose-2, 6-bisphosphate in MAECs isolated from *Prkaa1*$^{f/f}$ and *Prkaa1*$^{VEC\text{-}KO}$ mice under 25% EGM-2 12h treatment. n = 12. **g.** The measurement of extracellular lactate level in HUVECs transfected with si*Ctrl* and si*PRKAA1* for 48h. n = 5. All data were expressed as mean ± SEM. Statistical significance was determined by unpaired Student's *t* test.* $p < 0.05$ was considered significant, ** $p < 0.01$, *** $p < 0.001$.

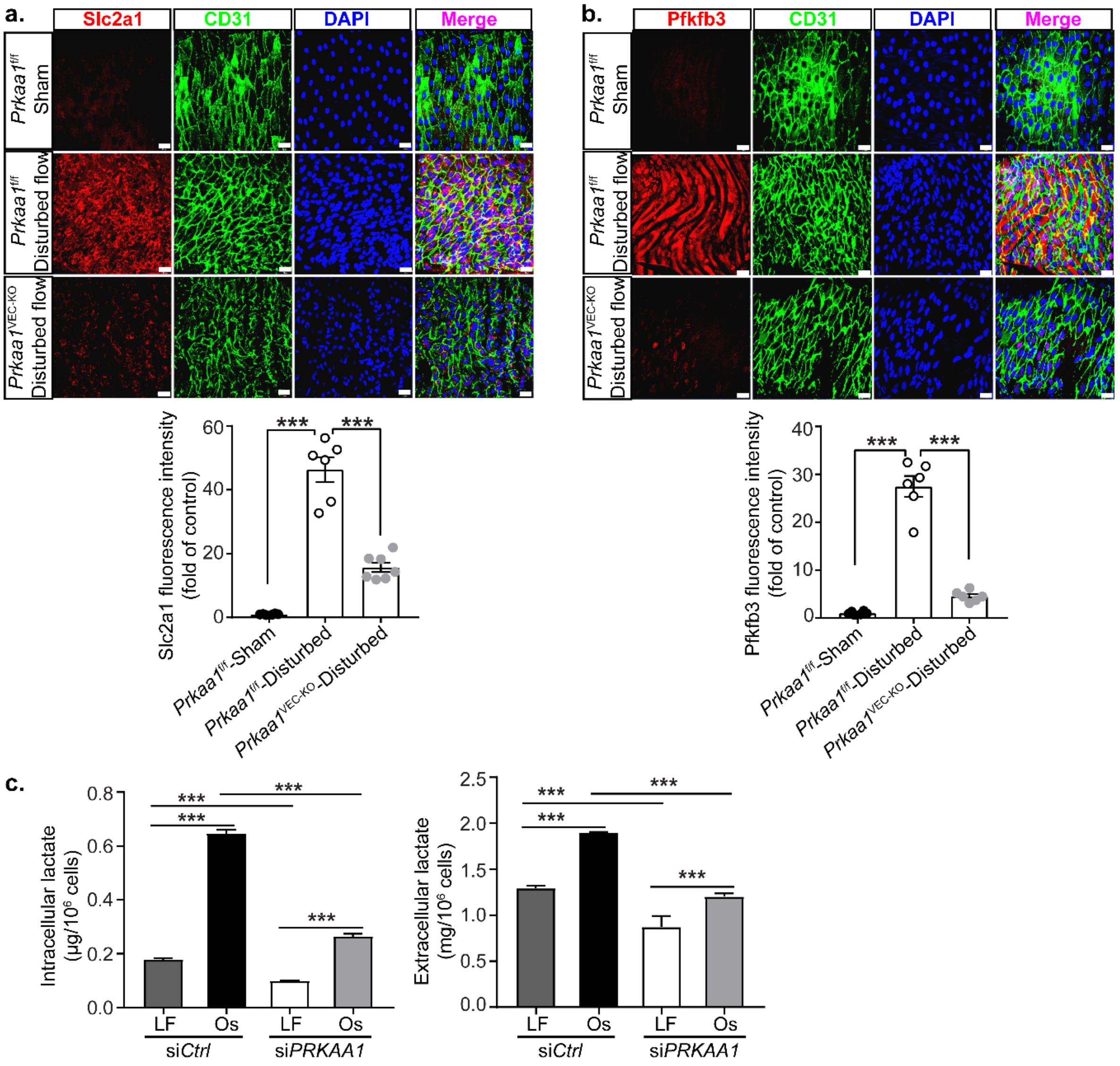

Supplementary Figure 3. PRKAA1 is required for disturbed flow-induced metabolic alterations of ECs. a-b. *En face* immunofluorescence staining and quantification of Slc2a1 and Pfkfb3 in sham-operated and partially ligated left common carotid arteries in *Prkaa1*$^{f/f}$ and *Prkaa1*$^{VEC\text{-}KO}$ mice co-stained with CD31 and DAPI. Scale bar: 20 μm; n = 6-7 mice per group. **c.** Intracellular and extracellular lactate levels in HUVECs transfected with si*Ctrl* and si*PRKAA1* under laminar flow and oscillating flow for 24h. n = 5. All data were expressed as mean ± SEM. Statistical significance was determined by one-way ANOVA followed by Bonferroni test. * $p < 0.05$ was considered significant, ** $p < 0.01$, *** $p < 0.001$.

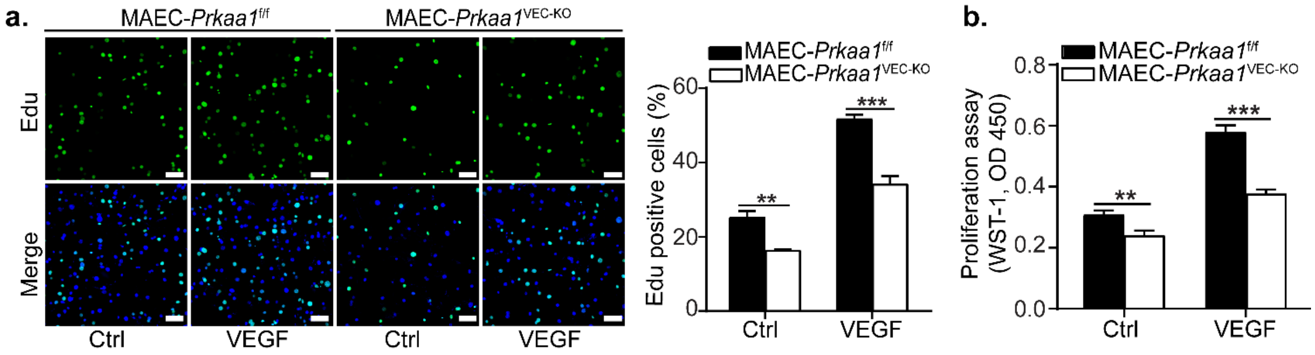

Supplementary Figure 4. Loss of Prkaa1 decreases endothelial proliferation. a. Representative images and quantification data of Edu staining of MAECs isolated from *Prkaa1*$^{f/f}$ and *Prkaa1*$^{VEC\text{-}KO}$ mice under 25% EGM-2 and VEGF 20ng/ml 12h treatment. Scale bar: 100 µm. n = 5. **b.** Cell proliferation of MAECs isolated from *Prkaa1*$^{f/f}$ and *Prkaa1*$^{VEC\text{-}KO}$ mice under 25% EGM-2 and VEGF 20ng/ml 12h treatment measured by WST-1 cell proliferation assay. n = 9. All data were expressed as mean ± SEM. Statistical significance was determined by unpaired Student's *t* test. * $p < 0.05$ was considered significant, ** $p < 0.01$, *** $p < 0.001$.

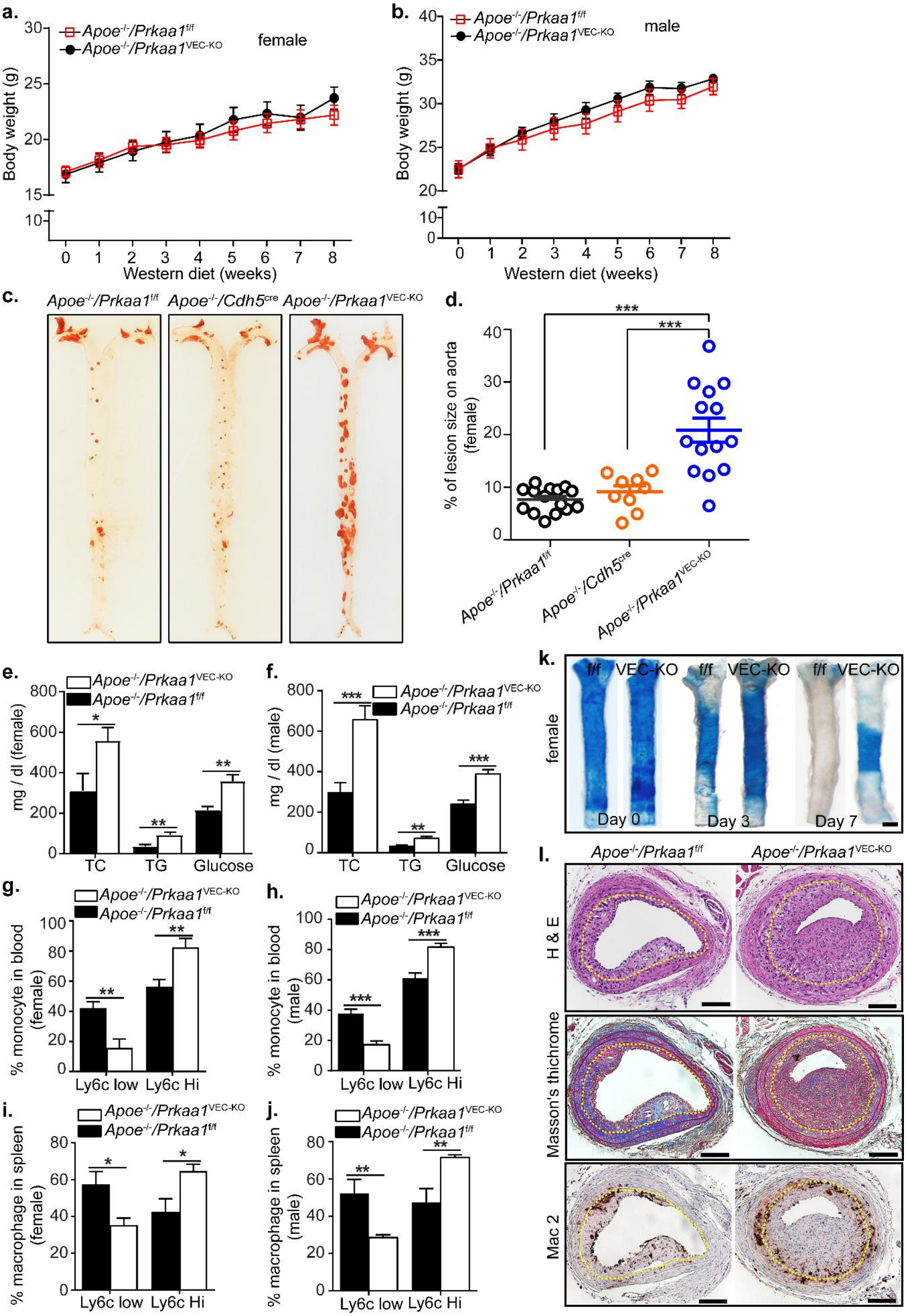
a.
Apoe-/-/Prkaa1f/f
Apoe-/-/Prkaa1VEC-KO
female
Body weight (g)
Western diet (weeks)
b.
Apoe-/-/Prkaa1f/f
Apoe-/-/Prkaa1VEC-KO
male
Body weight (g)
Western diet (weeks)
c.
Apoe-/-/Prkaa1f/f
Apoe-/-/Cdh5cre
Apoe-/-/Prkaa1VEC-KO
d.
% of lesion size on aorta (female)
Apoe-/-/Prkaa1f/f
Apoe-/-/Cdh5cre
Apoe-/-/Prkaa1VEC-KO
e.
Apoe-/-/Prkaa1VEC-KO
Apoe-/-/Prkaa1f/f
mg / dl (female)
TC
TG
Glucose
f.
Apoe-/-/Prkaa1VEC-KO
Apoe-/-/Prkaa1f/f
mg / dl (male)
TC
TG
Glucose
g.
Apoe-/-/Prkaa1VEC-KO
Apoe-/-/Prkaa1f/f
% monocyte in blood (female)
Ly6c low
Ly6c Hi
h.
Apoe-/-/Prkaa1VEC-KO
Apoe-/-/Prkaa1f/f
% monocyte in blood (male)
Ly6c low
Ly6c Hi
i.
Apoe-/-/Prkaa1VEC-KO
Apoe-/-/Prkaa1f/f
% macrophage in spleen (female)
Ly6c low
Ly6c Hi
j.
Apoe-/-/Prkaa1VEC-KO
Apoe-/-/Prkaa1f/f
% macrophage in spleen (male)
Ly6c low
Ly6c Hi
k.
f/f
VEC-KO
female
Day 0
Day 3
Day 7
l.
Apoe-/-/Prkaa1f/f
Apoe-/-/Prkaa1VEC-KO
H & E
Masson's thichrome
Mac 2

Supplementary Figure 5. Loss of endothelial *Prkaa1* increases lesion burden in *Apoe*$^{-/-}$ mice and accelerates neointima formation. a-b. Body weight of *Apoe*$^{-/-}$*/Prkaa1*$^{f/f}$ and *Apoe*$^{-/-}$*/Prkaa1*$^{VEC\text{-}KO}$ female and male mice fed Western-diet for 8 weeks. n = 8. **c.** Representative images of Oil Red O-stained aortas (*en face*) from *Apoe*$^{-/-}$*/Prkaa1*$^{f/f}$ (female n = 15), *Apoe*$^{-/-}$ *Cdh5*^{cre} (female n = 9), *Apoe*$^{-/-}$*/Prkaa1*$^{VEC\text{-}KO}$ (female n = 15) mice after 16 weeks of Western diet. **d.** Lesion area quantification data. **e-f.** Levels of total cholesterol (TC), triglyceride (TG), and glucose in *Apoe*$^{-/-}$*/Prkaa1*$^{f/f}$ (female, n = 6; male, n = 8), *Apoe*$^{-/-}$ *Prkaa1*$^{VEC\text{-}KO}$ (female, n = 6; male, n = 8) mice fed with 16 weeks of Western diet. **g-j.** Flow cytometry analysis of LyC6^{hi} monocytes or macrophages in circulating blood and spleen from *Apoe*$^{-/-}$*/Prkaa1*$^{f/f}$ (female, n = 6; male, n = 6), *Apoe*$^{-/-}$*/Prkaa1*$^{VEC\text{-}KO}$ (female, n = 6; male, n = 6) mice fed with 16 weeks of Western diet. **k.** Representative images of Evans blue staining of injured carotid arteries harvested at the indicated time points in *Prkaa1*$^{f/f}$ and *Prkaa1*$^{VEC\text{-}KO}$ female mice. Scale bar: 500 μm. **l.** Representative images of paraffin cross-sections of wire-injured carotid artery from *Apoe*$^{-/-}$*/Prkaa1*$^{f/f}$, *Apoe*$^{-/-}$*/Prkaa1*$^{VEC\text{-}KO}$ female mice with 4 weeks of Western diet stained with hematoxylin and eosin, Masson's trichrome, and Mac 2. Scale bar: 100 μm. All data were expressed as mean ± SEM. Statistical significance was determined by unpaired Student's *t* test (for **e-j**) and one-way ANOVA followed by Bonferroni test (for **d**). * $p < 0.05$ was considered significant, ** $p < 0.01$, *** $p < 0.001$.

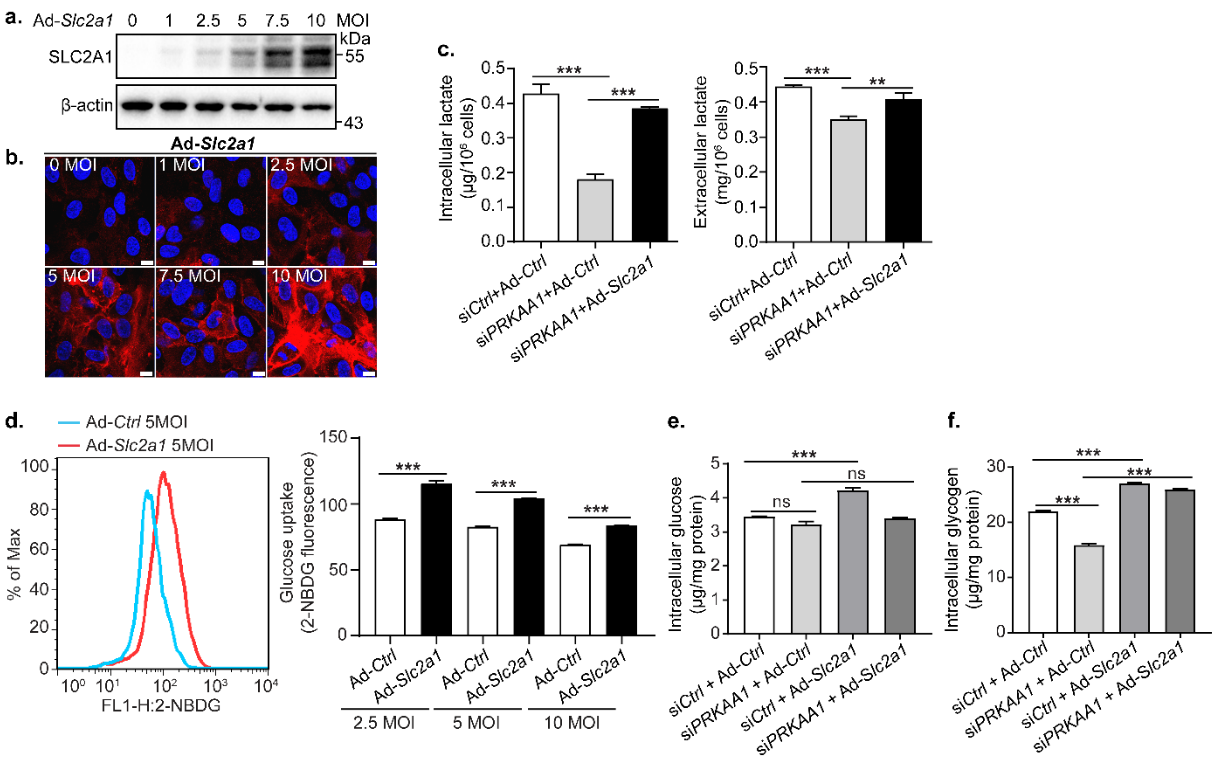

Supplementary Figure 6. Adenovirus transduction in HUVECs. a. Western-blot analysis of SLC2A1 protein level in HUVECs transfected with different doses of *Slc2a1*-overexpressing adenovirus (Ad-*Slc2a1*) for 48h. **b.** Immunofluorescence staining of SLC2A1 protein level in HUVECs transfected with different doses of *Slc2a1*-overexpressing adenovirus (Ad-*Slc2a1*) for 48h. Scale bar: 10 µm; n = 3. **c.** Intracellular and extracellular lactate levels in HUVECs transfected with si*Ctrl*-Ad-*ctrl*, si*PRKAA1*-Ad-*ctrl* and si*PRKAA1*-Ad-*Slc2a1* for 48h. n = 6. **d.** Representative images and quantification data of flow cytometry analysis of 2-NBDG staining in HUVECs transfected with different doses of Ad-*Slc2a1* for 48h. n = 5. **e-f.** The levels of intracellular glucose and glycogen in HUVECs transfected with si*Ctrl*-Ad-*Ctrl*, si*PRKAA1*-Ad-*Ctrl*, si*Ctrl*-Ad-*Slc2a1* and si*PRKAA1*-Ad-*Slc2a1* for 48h. n = 4. Ad-*Ctrl* and Ad-*Slc2a1* at 5 MOI were used in **c**, **e** and **f**. All data were expressed as mean ± SEM. Statistical significance was determined by unpaired Student's *t* test (for **d**) and one-way ANOVA followed by Bonferroni test (for **c**, **e**, **f**). * $p < 0.05$ was considered significant, ** $p < 0.01$, *** $p < 0.001$.

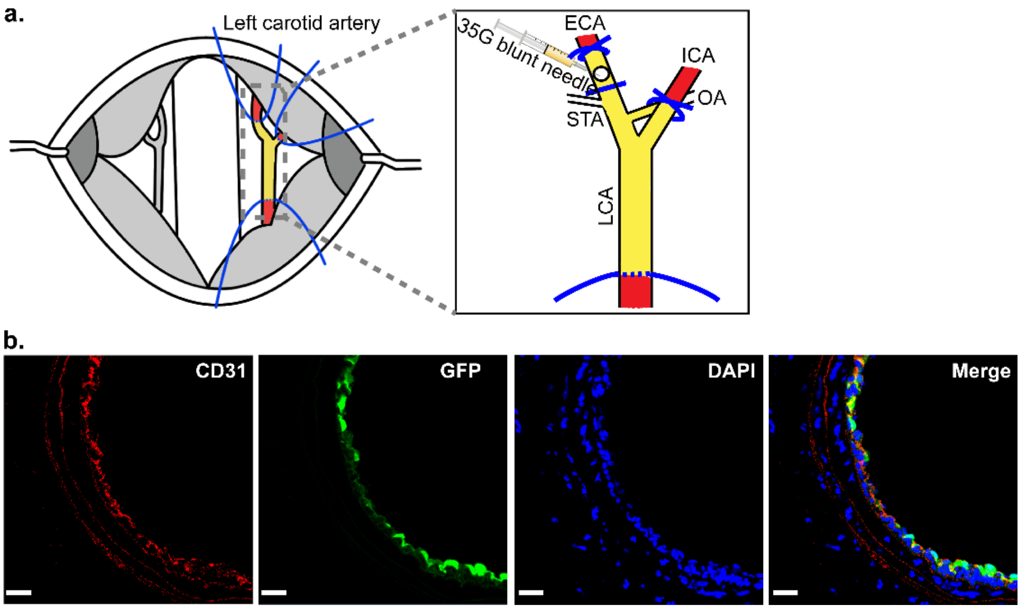

Supplementary Figure 7. Adenovirus transduction in mouse carotid arteries. a. Schematic illustration of mouse carotid arteries transfected with Ad-*Ctrl* and Ad-*Slc2a1* in partial ligation model. **b.** Representative images of CD31 (red) and GFP (green) staining in mouse carotid arteries transfected with Ad-*GFP* followed by (a) method. Scale bar: 20 μm.

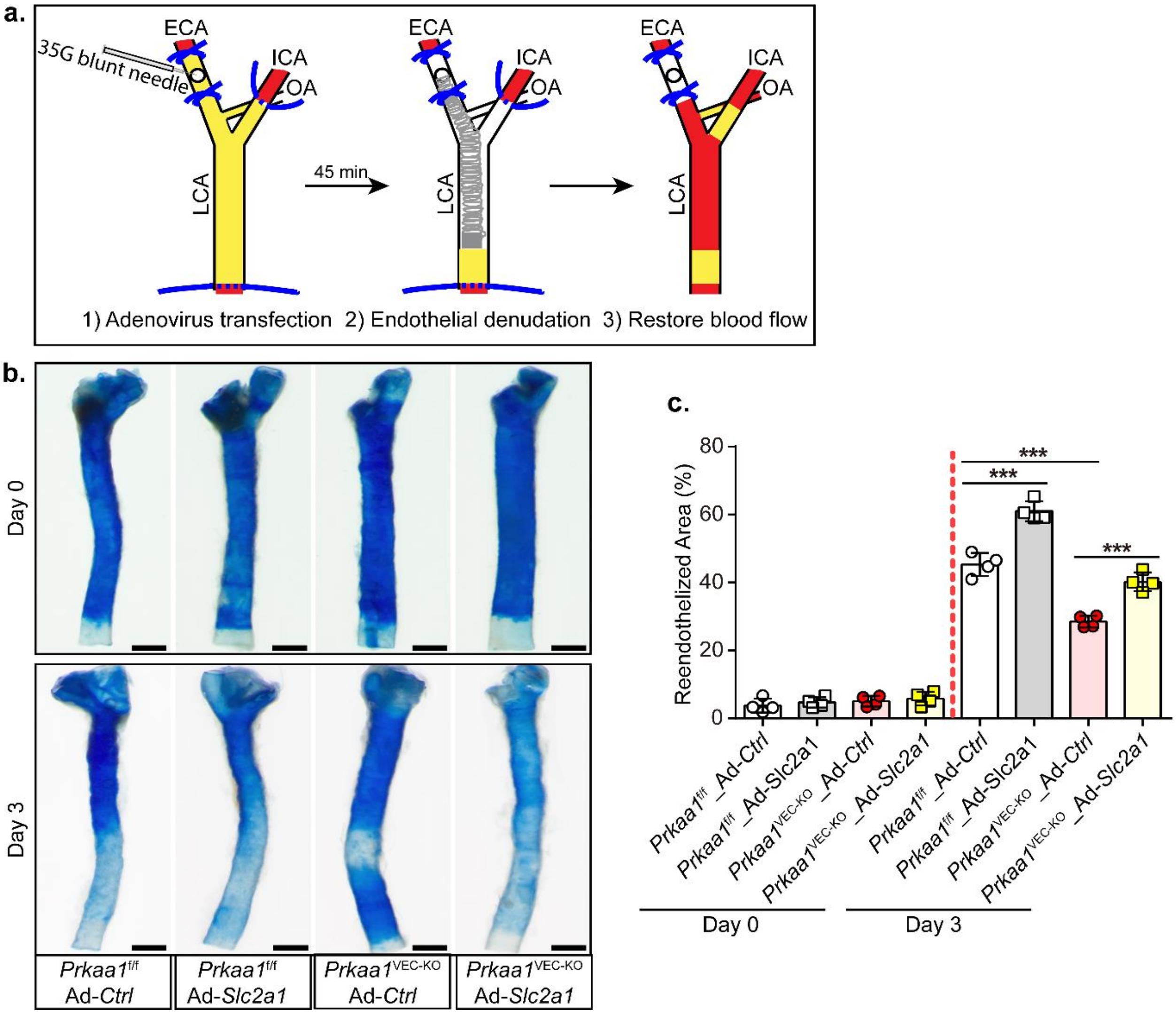

Supplementary Figure 8. Transduction of the endothelium with Ad-*Slc2a1* accelerated reendothelialization. a. Schematic illustration of mouse carotid arteries transfected with Ad-*Ctrl* and Ad-*Slc2a1* followed by endothelial denudation in partial ligation model. **b.** Representative images of Evans blue staining of injured carotid arteries harvested at the indicated time points in *Prkaa1*$^{f/f}$ and *Prkaa1*$^{VEC-KO}$ mice. Scale bar: 500 µm. **c.** Quantification data of percentage of reendothelialization over time in the injured carotid artery from *Prkaa1*$^{f/f}$ and *Prkaa1*$^{VEC-KO}$ mice. n = 4, for each time point. All data were expressed as mean ± SEM. Statistical significance was determined by one-way ANOVA followed by Bonferroni test. * $p < 0.05$ was considered significant, ** $p < 0.01$, *** $p < 0.001$.

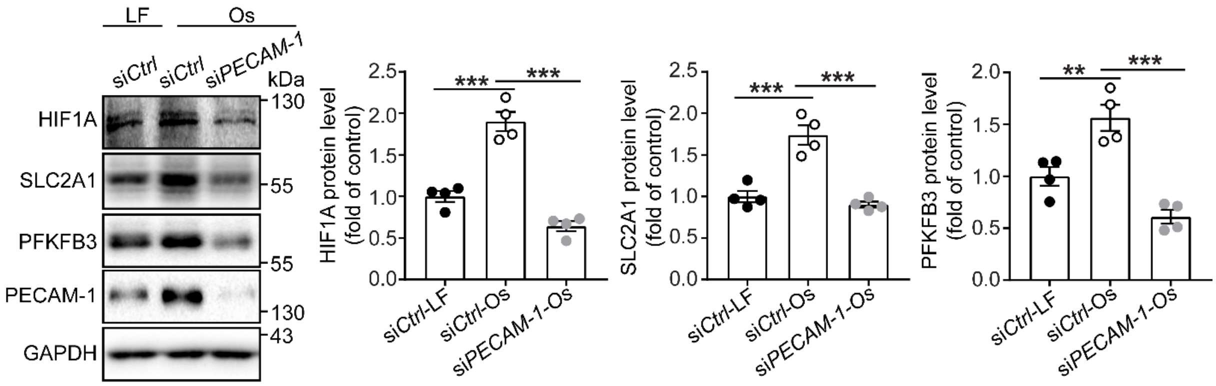

Supplementary Figure 9. PECAM-1 affects oscillating flow-mediated endothelial glycolysis. Western-blot analysis and quantification data of protein levels of HIF1A, SLC2A1 and PFKFB3 in HUVECs transfected with si*Ctrl* and si*PECAM-1* under laminar and oscillating flow for 24h. n = 4. All data were expressed as mean ± SEM. Statistical significance was determined by one-way ANOVA followed by Bonferroni test. * $p < 0.05$ was considered significant, ** $p < 0.01$, *** $p < 0.001$.

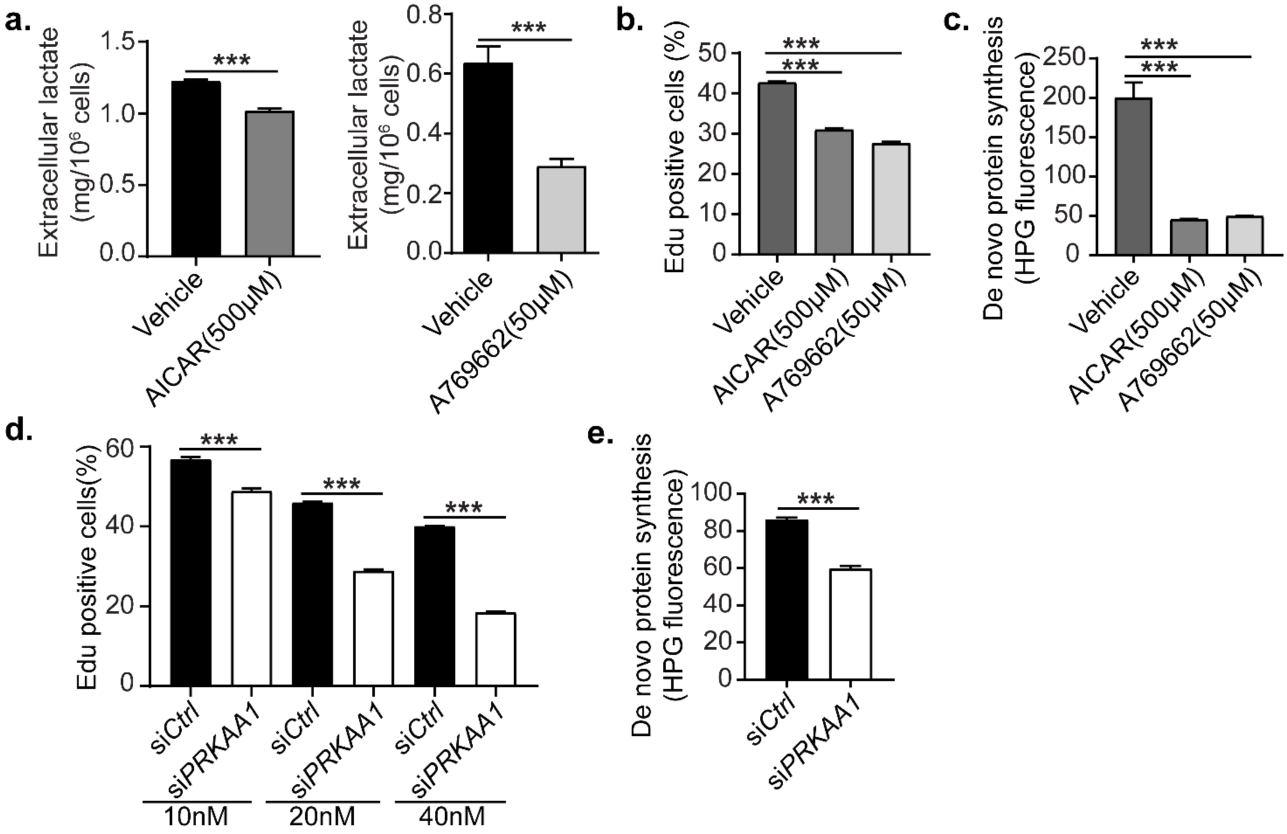

Supplementary Figure 10. The effect of *PRKAAA1* knockdown or activation on endothelial protein synthesis and proliferation. a. The extracellular lactate levels of HUVECs treated with 500 μM AICAR, 50 μM A769662 and vehicle treatment for 12h. n = 5. **b, c.** Proliferation and *de novo* protein synthesis in HUVECs treated with 500 μM AICAR, 50 μM A769662 and vehicle treatment for 12h. n = 5. **d.** Quantification data of flow cytometry analysis of Edu staining (10 μM, 12h) in HUVECs transfected with si*Ctrl* and si*PRKAA1* at different doses. n = 5. **e.** *De novo* protein synthesis in HUVECs transfected with si*Ctrl* and si*PRKAA1* (20 nM) for 48h. n = 5. HPG, L-homopropargylglycine. All data were expressed as mean ± SEM. Statistical significance was determined by unpaired Student's *t* test (for **a**, **d**, **e**) and one-way ANOVA followed by Bonferroni test (for **b** and **c**). * $p < 0.05$ was considered significant, ** $p < 0.01$, *** $p < 0.001$.

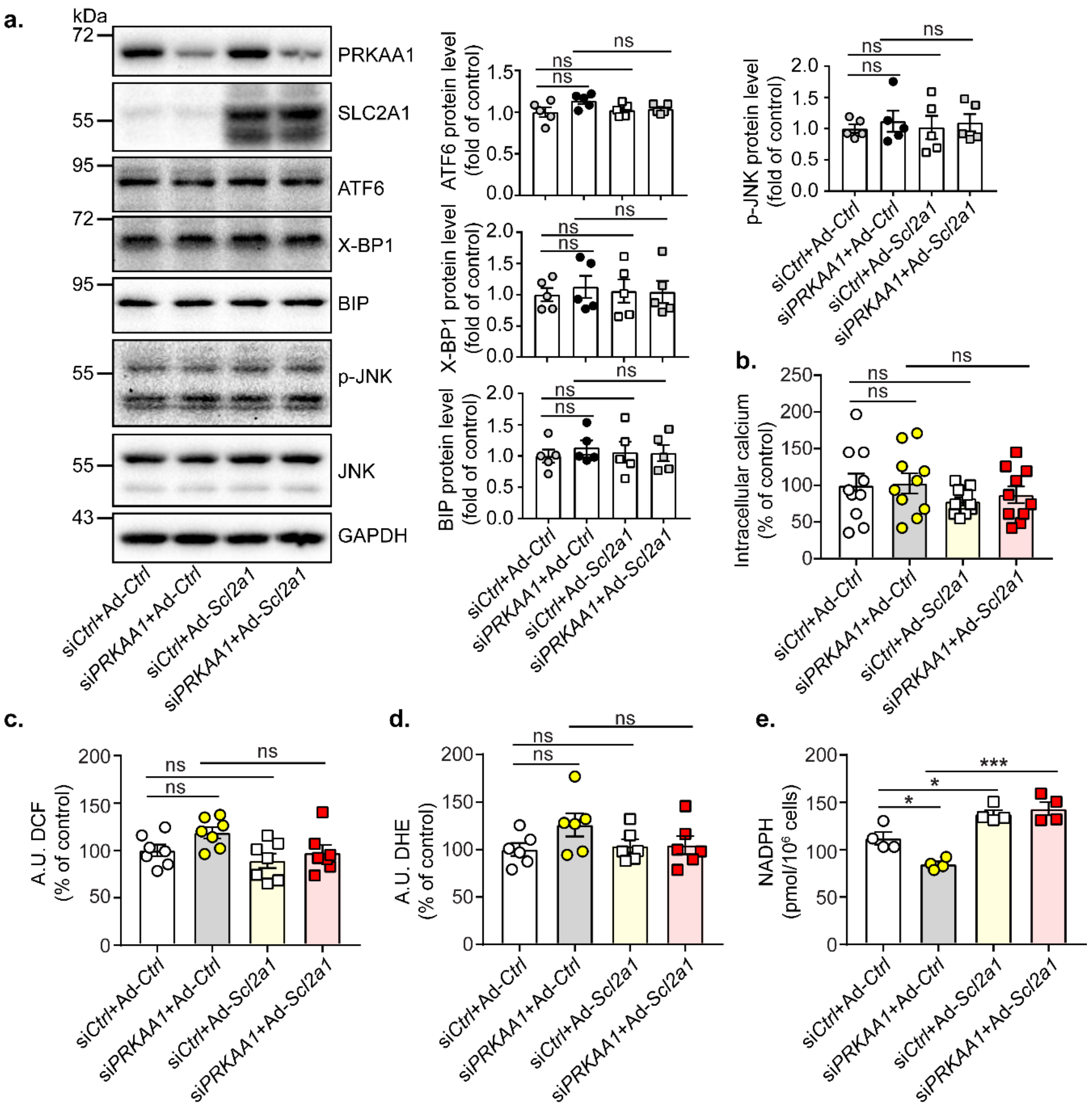
a.
kDa
PRKAA1
SLC2A1
ATF6
X-BP1
BIP
p-JNK
JNK
GAPDH
siCtrl+Ad-Ctrl
siPRKAA1+Ad-Ctrl
siCtrl+Ad-Scl2a1
siPRKAA1+Ad-Scl2a1
ATF6 protein level (fold of control)
X-BP1 protein level (fold of control)
BIP protein level (fold of control)
p-JNK protein level (fold of control)
ns
b.
Intracellular calcium (% of control)
c.
A.U. DCF (% of control)
d.
A.U. DHE (% of control)
e.
NADPH (pmol/10⁶ cells)
*
***

Supplementary Figure 11. The levels of ER stress, calcium and ROS in *PRKAA1* KD or *Slc2a1* overexpressing HUVECs. a. Western-blot analysis and quantification data of protein levels of ATF6, X-BP1, BIP, p-JNK and JNK in HUVECs transfected with si*Ctrl*-Ad-*Ctrl*, si*PRKAA1*-Ad-*Ctrl*, si*Ctrl*-Ad-*Slc2a1* and si*PRKAA1*-Ad-*Slc2a1* for 48h. n = 5. **b.** The level of intracellular calcium in HUVECs transfected with si*Ctrl*-Ad-*Ctrl*, si*PRKAA1*-Ad-*Ctrl*, si*Ctrl*-Ad-*Slc2a1* and si*PRKAA1*-Ad-*Slc2a1* for 48h. n = 10. **c, d, e.** The levels of ROS-DCF, ROS-DHE and NADPH in HUVECs transfected with si*Ctrl*-Ad-*Ctrl*, si*PRKAA1*-Ad-*Ctrl*, si*Ctrl*-Ad-*Slc2a1* and si*PRKAA1*-Ad-*Slc2a1* for 48h. n = 4-7. All data were expressed as mean ± SEM. Statistical significance was determined by unpaired Student's *t* test (for **g**) and one-way ANOVA followed by Bonferroni test. * $p < 0.05$ was considered significant, ** $p < 0.01$, *** $p < 0.001$.

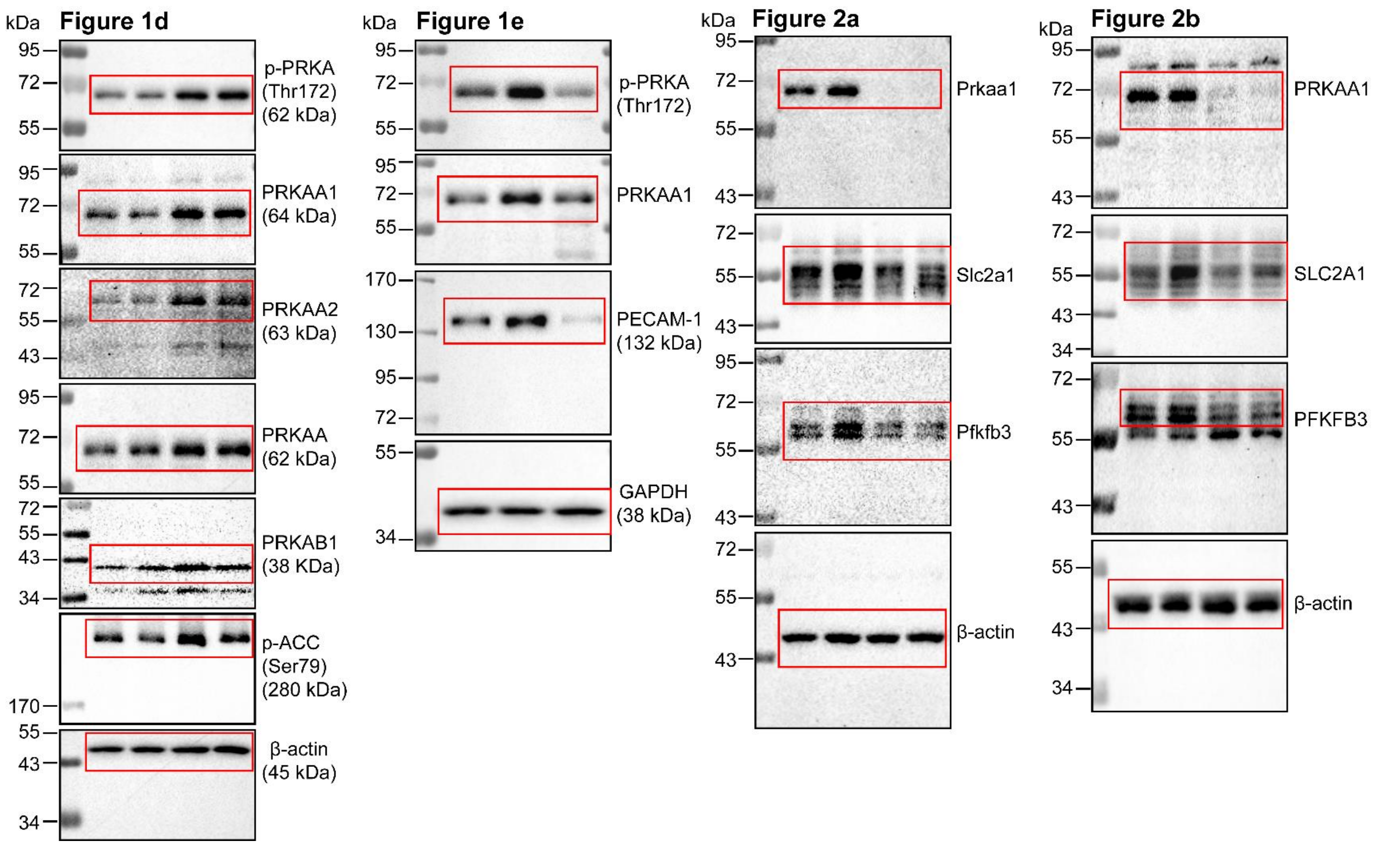

Supplementary Figure 12. Uncropped scans of the Western blots presented in Fig. 1d, 1e, 2a and 2b.

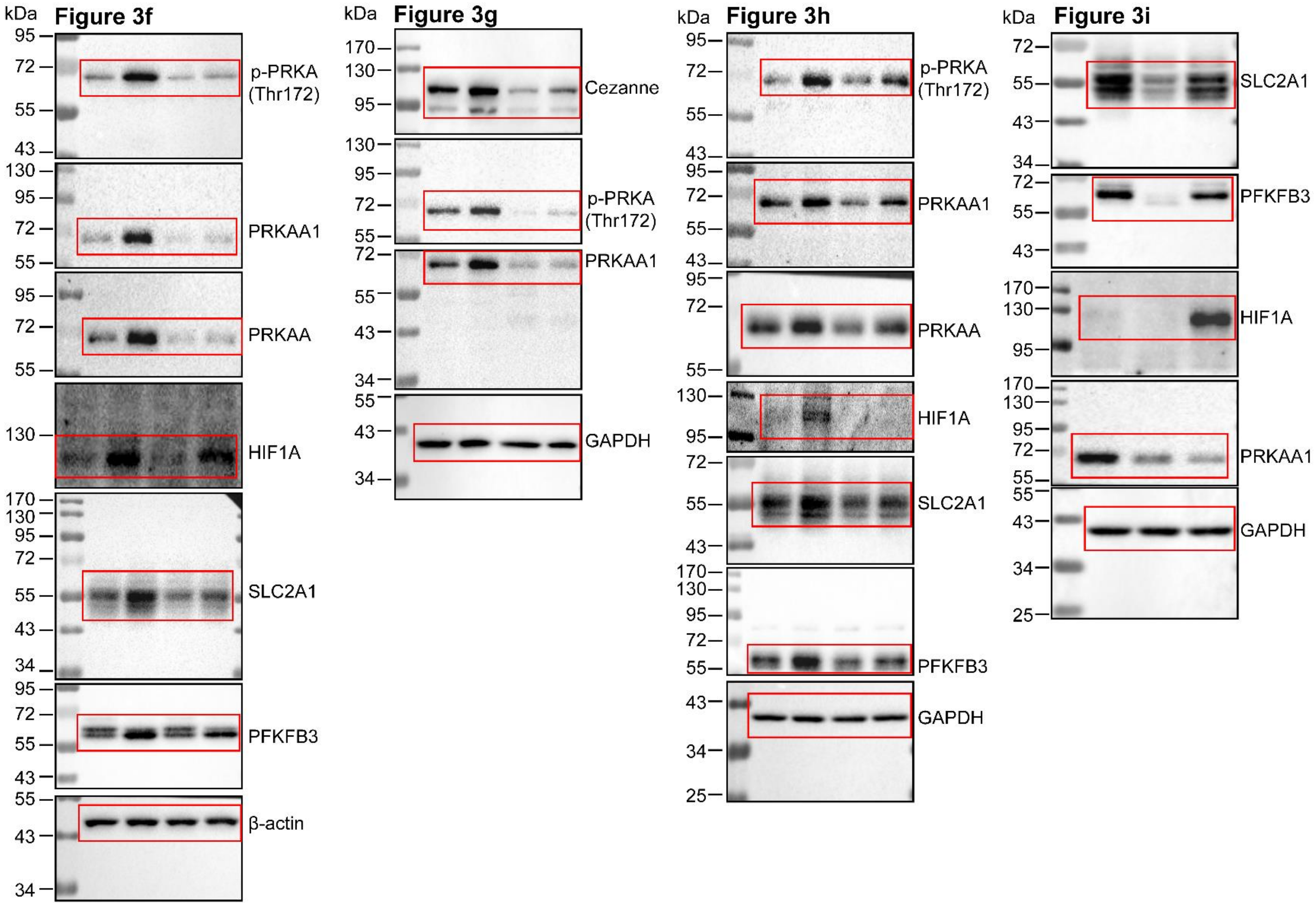

Supplementary Figure 13. Uncropped scans of the Western blots presented in Fig. 3f, 3g, 3h and 3i.

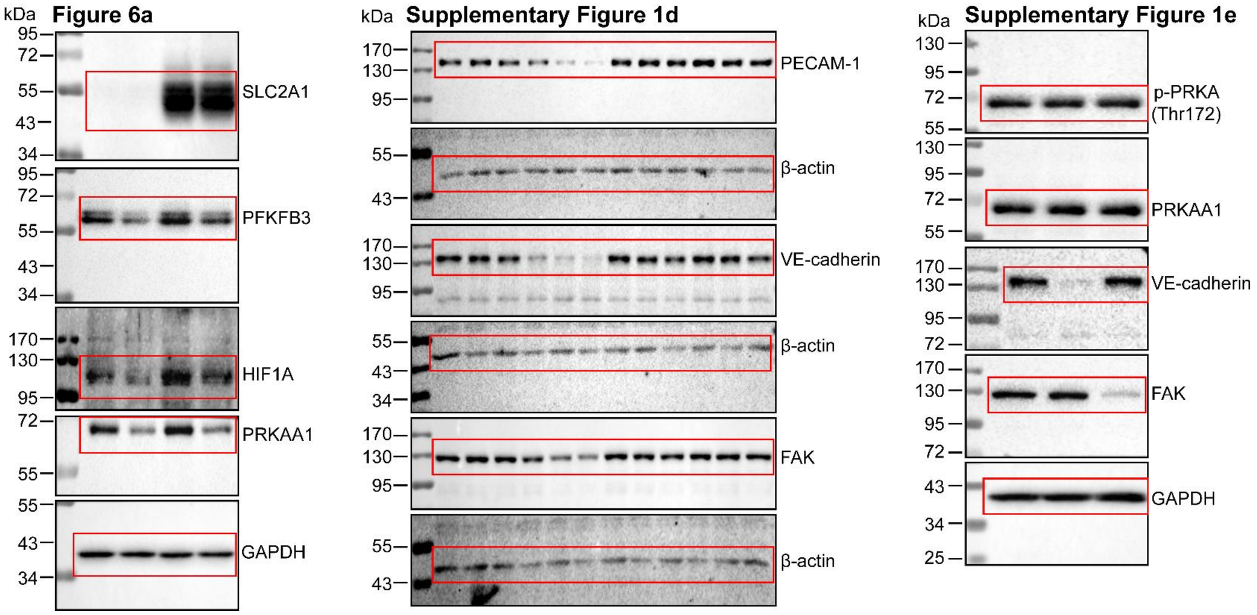

Supplementary Figure 14. Uncropped scans of the Western blots presented in Fig. 6a, Supplementary Fig. 1d and 1e.

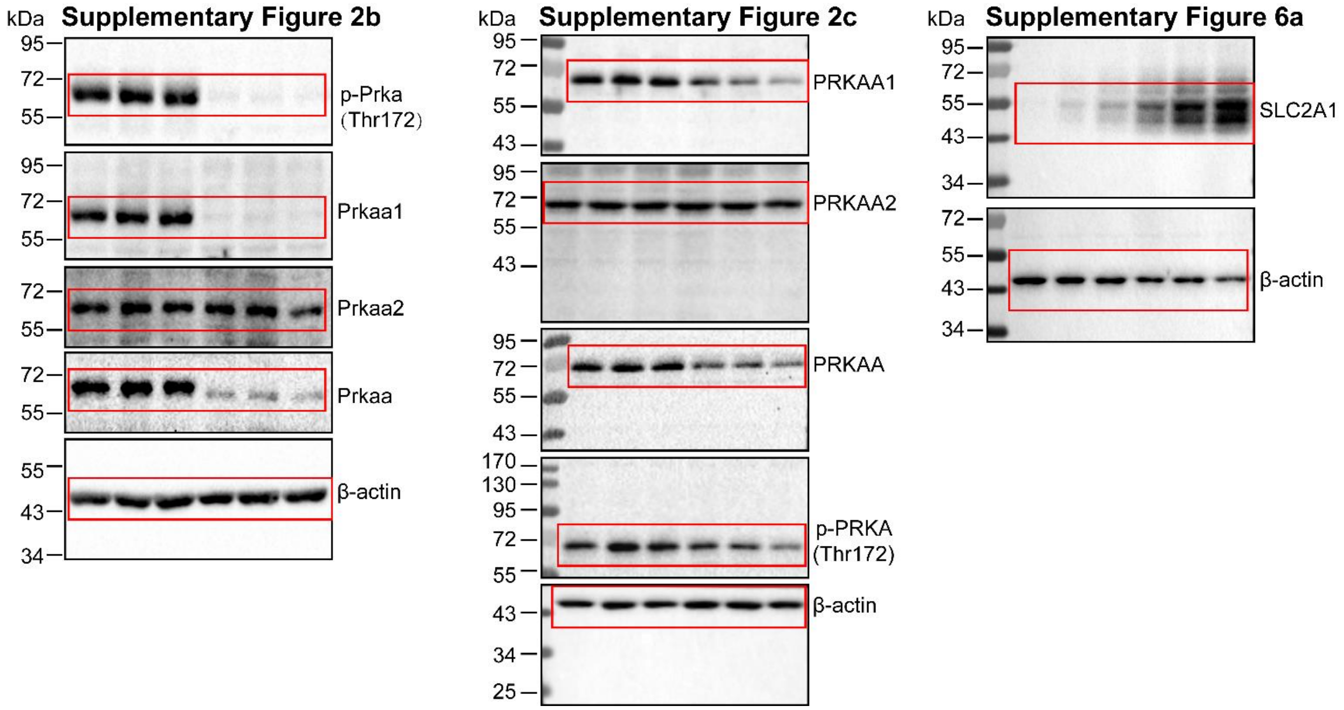

Supplementary Figure 15. Uncropped scans of the Western blots presented in Supplementary Fig. 2b, 2c and 6a.

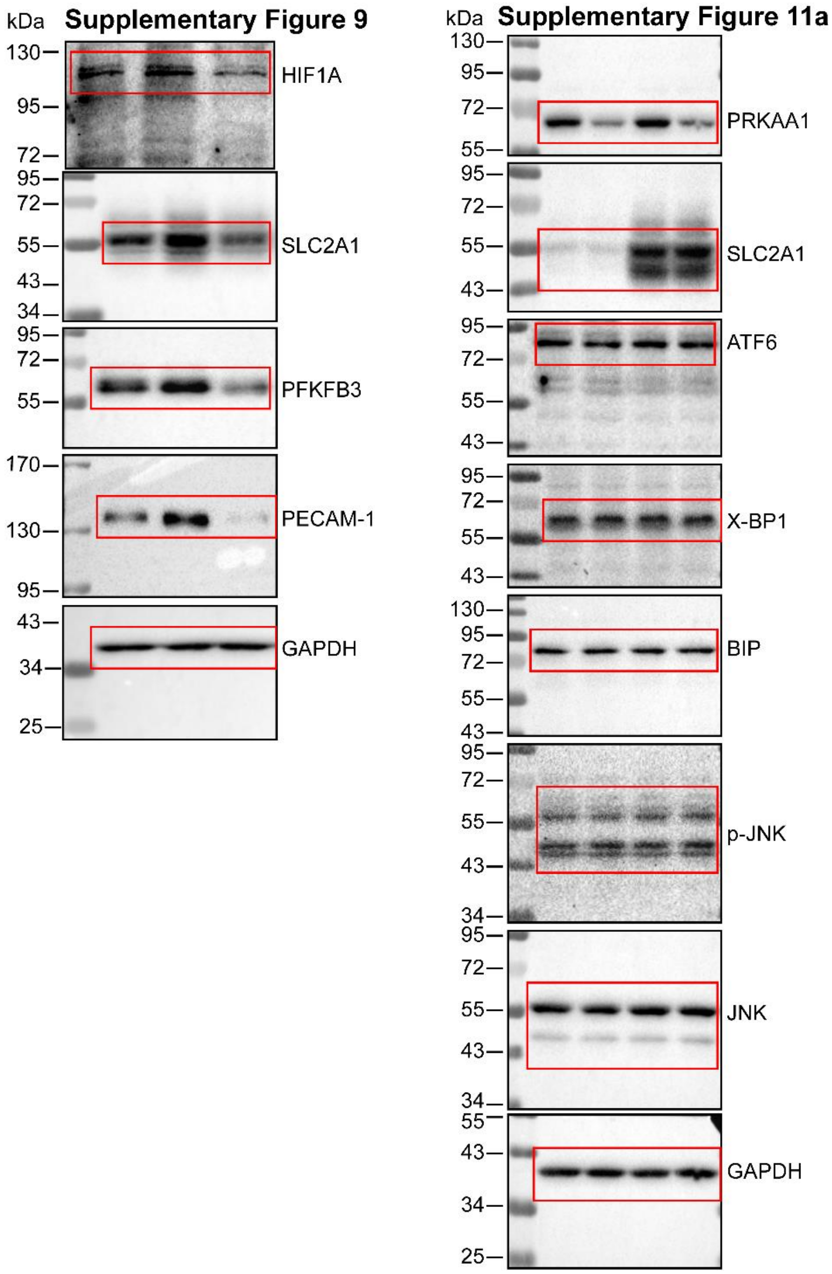

Supplementary Figure 16. Uncropped scans of the Western blots presented in Supplementary Fig. 9 and 11a.

Supplementary Tables

Supplementary Table 1. Primers used. Primers used for genotyping of murine strains and for real-time quantitative PCR

	FORWARD	REVERSE
**Genotyping**		
Prkaa1 flox	TATTGCTGCCATTAGGCTAC	GACCTGACAGAATAGGATATGCCC
Cdh5 Cre	GCGGTCTGGCAGTAAAAACTATC	GTGAAACAGCATTGCTGTCACTT
Apoe	GCCTAGCCGAGGGAGAGCCG	TGTGACTTGGGAGCTCTGCAGC
		GCCGCCCCGACTGCATCT
**RT-PCR**		
18s	CTTAGAGGGACAAGTGGCG	ACGCTGAGCCAGTCAGTGTA
murine *Prkaa1*	TGTCTCTGGAGGAGAGCTATTTGA	GGTGAGCCACAGCTTGTTCTT
murine *Prkaa2*	CAGAAGATTCGCAGTTTAGATGTTGT	ACCTCCAGACACATATTCCATTACC
murine *Prkab1*	*GTTGCTGTTGCTTGTTCCAA*	ATACTGTGCCTGCCTCTGCT
murine *Prkag1*	TCCCTAGACCTCACCACACC	GTCTGCACAGCACAAGAACC
murine *Hif1a*	TGCCCCAGATTCAAGATCAGC	TGCCCCAGATTCAAGATCAGC
murine *Slc2a1*	GCAGTTCGGCTATAACACTGG	GCGGTGGTTCCATGTTTGATTG
murine *Pfkfb3*	GATCTGGGTGCCCGTCGATCACCG	CAGTTGAGGTAGCGAGTCAGCTTC
murine *Hk1*	AACGGCCTCCGTCAAGATG	GCCGAGATCCAGTGCAATG
murine *Ldha*	CAAAGACTACTGTGTAACTGCGA	TGGACTGTACTTGACAATGTTGG
human *PRKAA1*	TCAGGAAGATTGTATGCAGGCCCA	TTCATGGGATCCACCTGCAGCATA
human *PRKAA2*	TTTGTGGCACCCTCCCATTTGATG	AGAACAGGAACGCTGAGGTGTTGA
human *PRKAB1*	TTTCGGGCACCCCCTATT	CCCGTGTCCTTGTTCAGGA
human *PRKAG1*	AGGGTGTTCTCAAGTGCTACCTG	CGGTGAACCTCTGCTTCCAC
human *HIF1A*	GAACGTCGAAAAGAAAAGTCTCG	CCTTATCAAGATGCGAACTCACA
human *SLC2A1*	GGCCAAGAGTGTGCTAAAGAA	ACAGCGTTGATGCCAGACAG
human *PFKFB3*	CTCGCATCAACAGCTTTGAGG	TCAGTGTTTCCTGGAGGAGTC
human *HK1*	GCTCTCCGATGAAACTCTCATAG	GGACCTTACGAATGTTGGCAA

Supplementary Table 2. Antibodies Used. Antibodies used for Western blotting, immunostaining, and FACS analysis.

Target protein	Species	Company	Catalog number #	Concentration Used	Molecular weight
**western blotting**					
p-PRKA (T172)	Rabbit	Cell Signaling	2531	1:1,000	62 KDa
PRKAA1	Mouse	Abcam	Ab110036	1:1,000	64 KDa
PRKAA2	Rabbit	Abcam	ab3760	1:1,000	63 KDa
PRKAB1	Rabbit	Cell Signaling	4150	1:1,000	38 KDa
PRKAA	Mouse	Abcam	Ab80039	1:1,000	62 KDa
p-ACC(S79)	Rabbit	Cell Signaling	3661	1:1,000	280 KDa
PFKFB3	Rabbit	Abcam	Ab181681	1:1,000	58 KDa
HIF1A	Goat	R&D systems	AF1935	1:500	120 KDa
SLC2A	Rabbit	Abcam	Ab115730	1:1,0000	40-60 KDa
β-actin	Mouse	Santa Cruz	sc-47778	1:5,000	45 KDa
PECAM-1	Mouse	Santa Cruz	sc-376764	1:1000	130 KDa
VE-cadherin	Mouse	R&D systems	MAB9381	1:1000	125 KDa
FAK	Rabbit	Cell Signaling	3285	1:1000	125 KDa
GAPDH	Mouse	Santa Cruz	sc-47724	1:5000	37 KDa
ATF6	Mouse	Active Motif	40962	1:1000	90 KDa
BIP	Rabbit	Cell Signaling	3177	1:1000	78 KDa
XBP-1	Rabbit	Cell Signaling	12782	1:1000	60 KDa
p-JNK1/2	Rabbit	Cell Signaling	4668	1:1000	46,54 KDa
JNK1/2	Rabbit	Cell Signaling	9258	1:1000	46,54 KDa
**Immunostaining**					
P-PRKA (T172)	Rabbit	GeneTex	GTX52341	1:100	
PRKAA1	Rabbit	GeneTex	GTX112998	1:200	
CD31	Rat	OPTISTAIN	DIA-310	1:100	
PFKFB3	Rabbit	Abcam	Ab181681	1:100	
SLC2A1	Rabbit	Abcam	Ab115730	1:100	
**FACS**					
F4/80	Rat	BD Bioscience	565410	1:100	PE
PE Rat IgG2a, κ Isotype Control	Rat	BD Bioscience	553930	1:100	PE
Ly6c	Rat	BD Bioscience	560595	1:100	APC
APC Rat IgG2a, κ Isotype Control	Rat	BD Bioscience	553932	1:100	APC
CD115	Rat	eBioscience	46-1152-82	1:100	PerCP
PerCP Rat IgG2a, κ Isotype Control	Rat	eBioscience	46-4321-80	1:100	PerCP
CD11b	Rat	eBioscience	46-0112-82	1:100	FITC
FITC Rat IgG2a, κ Isotype Control	Rat	BD Bioscience	553929	1:100	FITC

Supplementary Table 3. siRNA Used.

	**Target gene**	**Species**	**Company**	**Catalog number #**
siRNA	*PRKAA1*	Human	Santa Cruz Biotechnology	sc-29673
siRNA	Non-targeting	Human	Santa Cruz Biotechnology	sc-37007
siRNA	*HIF1A*	Human	Dharmacon	L-004018-00-0005 5 nmol
siRNA	*PECAM1*	Human	Dharmacon	L-017029-00-0005 5 nmol
siRNA	*CDH5*	Human	Dharmacon	L-003641-00-0005 5 nmol
siRNA	*PTK2*	Human	Dharmacon	L-003164-00-0005 5 nmol
siRNA	Non-targeting	Human	Dharmacon	D-001810-10-05
